# Candidate pheromone receptors of codling moth *Cydia pomonella* respond to pheromones and kairomones

**DOI:** 10.1038/srep41105

**Published:** 2017-01-24

**Authors:** Alberto Maria Cattaneo, Francisco Gonzalez, Jonas M. Bengtsson, Elizabeth A. Corey, Emmanuelle Jacquin-Joly, Nicolas Montagné, Umberto Salvagnin, William B. Walker, Peter Witzgall, Gianfranco Anfora, Yuriy V. Bobkov

**Affiliations:** 1 Fondazione Edmund Mach, Research and Innovation Centre/DASB, Agricultural Entomology, San Michele all’Adige, TN, Italy; 2 University of Florida, Whitney Laboratory, Center for Smell and Taste, and McKnight Brain Institute, Gainesville, FL, USA; 3 Università degli Studi di Milano, Department of Food, Nutritional and Environmental Sciences, Milan, Italy; 4 Università di Cagliari, Dipartimento di Scienze Biomediche, Monserrato, CA, Italy; 5 Swedish University of Agricultural Sciences, Department of Plant Protection Biology, Chemical Ecology Unit, Alnarp, Sweden; 6 Stockholm University, Department of Zoology, Stockholm, Sweden; 7 INRA, Institute of Ecology and Environmental Sciences (iEES-Paris), Versailles, France; 8 Sorbonne Universités, UPMC Univ Paris 06, iEES-Paris, Paris, France; 9 Max Planck Institute for Chemical Ecology, Department of Evolutionary Neuroethology, Jena, Germany

## Abstract

Olfaction plays a dominant role in the mate-finding and host selection behaviours of the codling moth (*Cydia pomonella*), an important pest of apple, pear and walnut orchards worldwide. Antennal transcriptome analysis revealed a number of abundantly expressed genes related to the moth olfactory system, including those encoding the olfactory receptors (ORs) CpomOR1, CpomOR3 and CpomOR6a, which belong to the pheromone receptor (PR) lineage, and the co-receptor (CpomOrco). Using heterologous expression, in both *Drosophila* olfactory sensory neurones and in human embryonic kidney cells, together with electrophysiological recordings and calcium imaging, we characterize the basic physiological and pharmacological properties of these receptors and demonstrate that they form functional ionotropic receptor channels. Both the homomeric CpomOrco and heteromeric CpomOrco + OR complexes can be activated by the common Orco agonists VUAA1 and VUAA3, as well as inhibited by the common Orco antagonists amiloride derivatives. CpomOR3 responds to the plant volatile compound pear ester ethyl-(E,Z)-2,4-decadienoate, while CpomOR6a responds to the strong pheromone antagonist codlemone acetate (E,E)-8,10-dodecadien-1-yl acetate. These findings represent important breakthroughs in the deorphanization of codling moth pheromone receptors, as well as more broadly into insect ecology and evolution and, consequently, for the development of sustainable pest control strategies based on manipulating chemosensory communication.

Tortricid moths (Lepidoptera: Tortricidae) are among the most important pest insects as a result of their economic impact on agriculture. Within this family, the codling moth *Cydia pomonella* (L.) is now found in apple, pear and walnut orchards worldwide. They rely on odour and pheromone perception to find food, mates, and suitable substrates for oviposition^1^,^2^, and semiochemical-based technologies have been used as part of sustainable control strategies.

Insect pheromones are detected by olfactory sensory neurons (OSNs) that innervate specialized cuticular sensilla, primarily found on the antennal surface. Odorant detection is mediated by specific olfactory receptors (ORs) working together with an olfactory co-receptor (Orco) as heteromeric ligand-gated ion channels^3^,^4^,^5^. While many ORs are tuned to environmental odours such as plant volatiles, pheromone receptors (PRs), which constitute a monophyletic clade in the insect OR phylogeny, respond predominantly to sex pheromones^6^,^7^,^8^,^9^. Previous transcriptome-based studies^10^,^11^, reported 58 putative codling moth ORs, of which 12 are grouped in the PR-clade. Deorphanization of these PRs may aid in attempts to increase the efficacy of pheromone-based mating disruption for this species.

The main component of the codling moth pheromone is (E,E)-8,10-dodecadien-1-ol (codlemone). Volatile compounds emitted from host-plants, such as pear ester, ethyl (E,Z)-2,4-decadienoate, are known to enhance male attraction to codling moth pheromone^9^,^12^,^13^,^14^. Disruption of mate-finding by air permeation with synthetic codlemone^2^,^15^ can accordingly be enhanced by adding pear ester^12^,^16^,^17^. Semiochemical-based technologies enable sustainable control of the insect model codling moth and can be applied also to other insects^18^.

In an attempt to identify the PRs responsible for detection of these ligands, we recently expressed CpomORs in *Drosophila melanogaster* OSNs by means of both the *Or67d*^GAL4^ line for expression in at1 OSNs housed in trichoid sensilla^19^, and the *Δ-halo Or22a-GAL4* line for expression in ab3A OSNs housed in basiconic sensilla^20^,^21^. Single sensillum recordings (SSR) from OSNs expressing one of the candidate PRs, CpomOR3, revealed that it did not respond to any of the pheromonal compounds emitted by *C. pomonella* females or closely related species within the genus *Cydia*^22^,^23^. Instead, it responds strongly to pear ester, which has a strong synergistic effect on male attraction to sex pheromone^9^,^24^,^25^.

Here we used HEK293T-based heterologous expression together with standard calcium imaging and patch-clamp recordings, as well as *in vivo* expression in *D. melanogaster* OSNs, for reinvestigation of CpomOR3 function and *de novo* characterization of two additional PR candidates, CpomOR1 and CpomOR6a, abundantly expressed in moth male OSNs and initially proposed to be possible receptors for codlemone^10^,^11^. We found that all three receptors are functional when co-expressed in HEK293T cells with CpomOrco. While we were unable to detect a response of CpomOR1 to any of the native ligands tested, CpomOR3 responded to pear ester as well as the analogous methyl-(E,Z)-2-4-decadienoate and CpomOR6a responded to codlemone acetate ((E,E)-8,10-dodecadien-1-yl acetate) a strong pheromone antagonist of the codling moth^26^. These findings represent important breakthroughs in understanding the mechanisms of codling moth attraction to biologically relevant odours and, consequently, for developing innovative pest control strategies based on disrupting olfactory communication.

## Results

### Codling moth putative PRs and Orco

**Figure 1:**
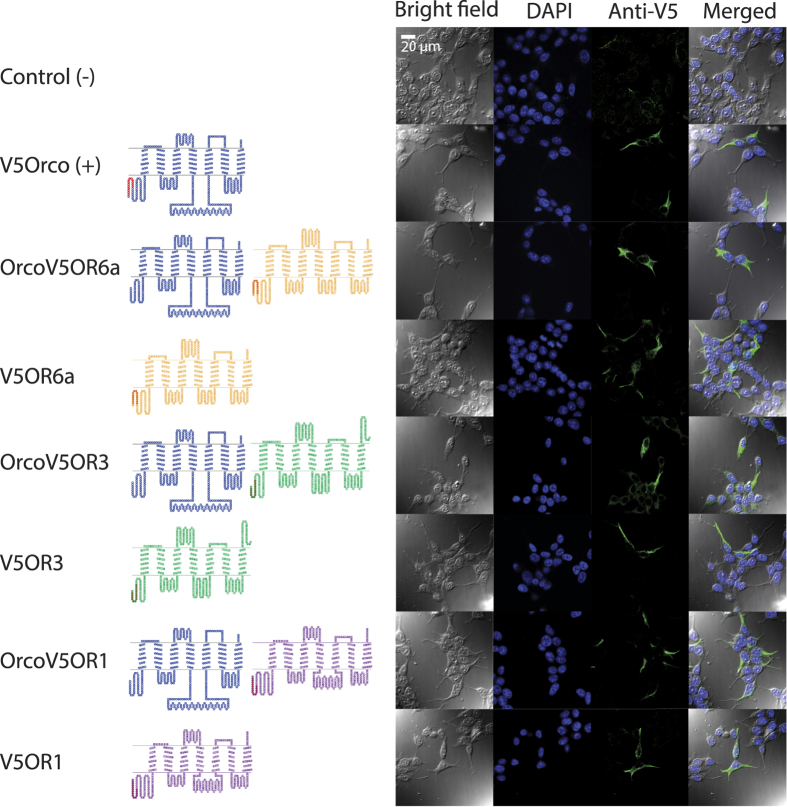
Expression and localization of OR channels in HEK293T cells and transmembrane topology of the receptors. Left: topological representation of codling moth olfactory receptors (TOPO 2.0): Blue: CpomOrco; Brown: CpomOR6a; Green: CpomOR3; Purple: CpomOR1; red residues: N-terminal V5 epitope tag (Nt-GKPIPNPLLGLDST-Ct). Right: Confocal microscopy analysis (bright field, DAPI, Anti-V5, Merged) of expression and localization of individual olfactory receptors or receptor complexes (as in left) in HEK293T cells. The significant fraction of the Anti-V5 antibody labelled targets can be localized to plasma membrane.

The complete open reading frames (ORFs) for CpomOR1 (JN836674.1), CpomOR3 (KJ420588), and CpomOrco (JN836672) were sequenced in prior studies^10^,^25^. For CpomOR6a, a 306 bp sequence was identified in the codling moth antennal transcriptome^10^. We performed 5′ RACE-PCR to complete the ORF of 1248 bp (updated to Genbank; accession number JN836671). Then, we compared the ORF sequence with the updated transcript variants found by Walker *et al*. (2016)^11^, and determined that this sequence corresponds to CpomOR6a. Analysing the translated protein, seven transmembrane domains with an intracellular localization for the N-terminus were predicted for CpomOR6a, CpomOR3, CpomOR1 and CpomOrco (Fig. 1), reflecting the typical topology of insect ORs^27^.

### Heterologous expression of codling moth putative PRs and Orco

Prior to physiological tests of the codling moth receptors in the HEK293T heterologous expression system, we first established that the receptors were expressed at the cell membrane. Each of the receptors was cloned into the mammalian expression vector pcDNA5/TO with an N-terminal V5 epitope tag. Twelve to 16 h after transfection, the cells were permeabilized and fixed for immunolabeling with an anti-V5 antibody. Untransfected cells were used as a negative control. Distinct labelling of the plasma membranes of transfected cells indicated that the receptors were properly expressed in the system (Fig. 1). The putative PRs were also co-expressed with CpomOrco, resulting in a localization similar to that found when they were expressed alone (Fig. 1).

### Physiological properties of the heterologously expressed codling moth putative PRs

**Figure 2:**
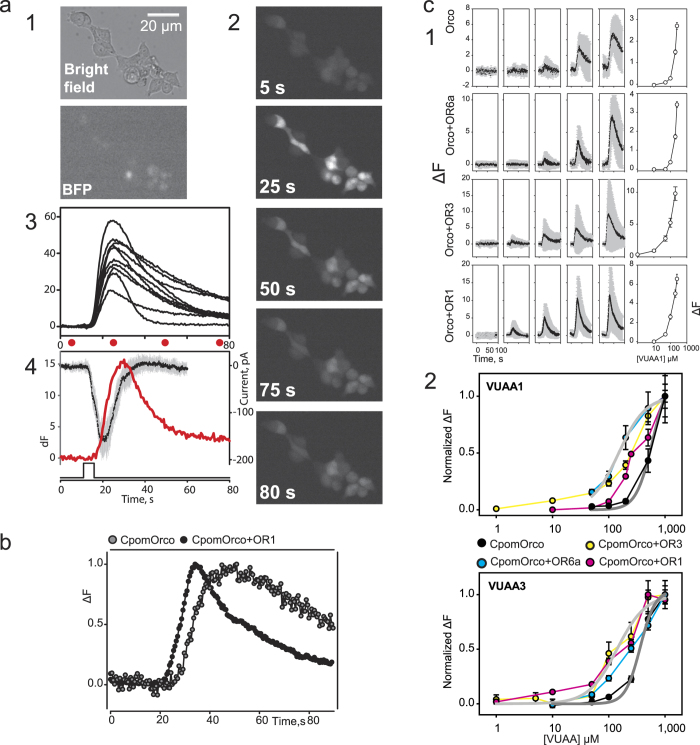
Electrophysiology and pharmacology of CpomORs expressed in HEK293T cells. **(a)** Activation of HEK293T cells expressing CpomOrco by VUAA1. CpomOrco was co-expressed with BFP (mostly localized in nuclei) for parallel expression control (a1); HEK293T cells were stimulated with VUAA1 (250 μM, a2). The agonist stimulation elicits Ca^++^_i_ increase in the cells (a2, a3). Each image (a2) was acquired at the times indicated on panels and by red circles under calcium signal curves (intensity of fluorescent curves, a3). Note: the BFP negative cells (compare bright field image with BFP and a2) did not generate appreciable calcium signal. (a4) Activation of ion channels underlies the generation of the agonist dependent calcium signal. Whole-cell voltage clamp recording was obtained from the cell that generated calcium signal in response to VUAAs (250 μM). VUAA1 activated inward current (black line, average of three responses, grey bars - SD) that kinetically preceded calcium signal (red line) and thus may underlie agonist dependent calcium influx. Holding potential was −50 mV. Time scale and diagram of stimulus application shown in a4 is common for a3 and a4. **(b)** The kinetics of calcium responses mediated by the activity of the homomeric Orco or Orco + OR complexes were different. Cells transfected with CpomOrco + OR1 could be characterized by faster activation/deactivation kinetics (black circles and line). Calcium response traces represent average of normalized responses of many cells recorded from the same preparation. **(c)** VUAA1 stimulation elicits dose-dependent Ca^++^_i_ increase in HEK293T cells expressing either homomeric Orco or Orco + OR complexes (plot series are labelled respectively). (c1) Data within each row were obtained from single preparation; right panels, the respective concentration dependences. Note: the maximal agonist concentration used in these experiments (VUAA1 250 μM) is likely not a saturating concentration. (c2) Concentration dependences of VUAA1 and VUAA3. Data were obtained in the separate series of experiments. The response amplitudes were used to generate the agonist concentration dependences. The average peak amplitudes of the responses of different cells (n = 57–216) were normalized to the maximal responses usually elicited by application of a saturating concentration (1000 μM) of VUAA1 or VUAA3. Note: homomeric CpomOrco (dark grey curves) is less sensitive to the agonists ([VUAA1]1/2 ~ 520 μM, [VUAA3]1/2 ~ 362 μM) than the heteromeric complexes (e.g. light grey curves, [VUAA1]1/2 ~ 150 μM, [VUAA3]1/2 ~ 140 μM).

The codling moth receptors were co-transfected with a plasmid carrying a gene for nucleus-targeted Blue Fluorescent Protein (BFP) as a control for transfection efficiency (Fig. 2). We began by testing the codling moth putative PRs coexpressed with Orco for functional expression using VUAA1 (acetamide,*N*-(4-ethylphenyl)-2-[[4-ethyl-5-(3-pyridinyl)-4*H*-1,2,4-triazol-3-yl]thio]-) or VUAA3 (acetamide,2-[[4-ethyl-5-(4-pyridinyl)-4*H*-1,2,4-triazol-3-yl]thio]-*N*-[4-(1-methylethyl)phenyl]-) agonists that are known to activate both heteromeric and homomeric Orco-containing receptor channels from other insect species^28^. HEK293T cells transfected with CpomOrco alone or CpomOrco in combination with either CpomOR6a, CpomOR3 or CpomOR1 generated calcium signals upon application of VUAA1 or VUAA3 (Fig. 2). Neither un-transfected nor transfected cells were sensitive to the maximum solvent concentration used (DMSO, 1%), suggesting that the agonist effects were not solvent dependent. Additionally, the overall sensitivity of the cells to the agonists correlated with BFP expression (Fig. 2). In the initial series of experiments, we examined whether the activation of ion channels underlies the generation of the agonist dependent calcium signals (Fig. 2). Whole-cell voltage clamp mode was used to record from cells sensitive to VUAAs. As shown for one cell (Fig. 2), at the holding potential −50 mV, VUAA1 activated an inward current (black line) that kinetically preceded the calcium signal (red line) and thus may determine the agonist mediated calcium influx. Interestingly, the kinetics of the calcium responses mediated by the activity of either homomeric CpomOrco or heteromeric CpomOrco + OR complexes were different (Fig. 2). For example, cells transfected with CpomOrco + OR1 could be characterized by faster activation/deactivation kinetics (Fig. 2). Similar results were observed for the two other heteromeric complexes: CpomOrco + OR6a and CpomOrco + OR3 (Fig. 2).

The response amplitude of both individual cells and cell populations depended on the agonist concentration. The population (cumulative) responses were used to generate the agonist concentration dependences (Fig. 2). For quantitative analysis and comparison, the average peak amplitudes of the responses of different cells were normalized to the responses elicited by application of a saturating concentration (1000 μM) of VUAA1 or VUAA3 (Fig. 2). The comparison of the dose-response characteristics obtained for different OR complexes suggests that homomeric CpomOrco is less sensitive to these agonists than its heteromeric counterparts are. Furthermore, the parameters of the calcium responses mediated by the activity of heteromeric CpomOrco + OR complexes are characterized by faster activation/deactivation kinetics (Fig. 2).

**Figure 3:**
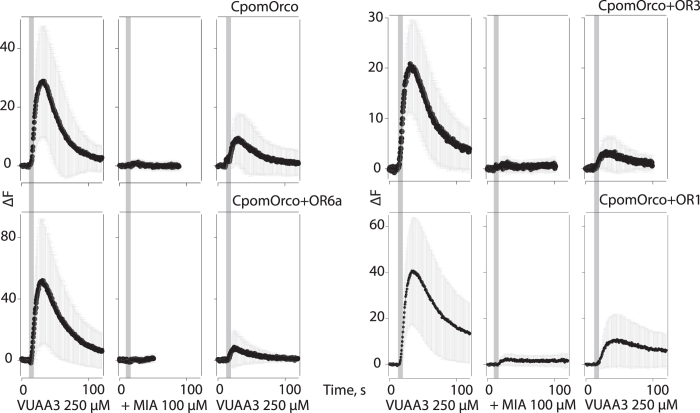
Sensitivity of CpomORs to amiloride derivatives when expressed in HEK293T cells. Amiloride derivative, MIA, 100 μM, applied extracellularly completely blocked the VUAA3 activated calcium signal. Similar results were observed for both homomeric (top-left panel) and all heteromeric complexes. Note: the effects were only partially reversible.

Amiloride derivatives (ADs) have been shown to block both heteromeric and homomeric currents during VUAA1 activation^29^,^30^. In our experiments with the putative Cpom PRs and Orco, the AD 5-(N-methyl-N-isobutyl) amiloride (MIA, 100 μM) applied extracellularly almost completely blocked the VUAA3 activated calcium signal (Fig. 3). Similar results were observed for both homomeric and all heteromeric complexes. In all cases, the effects were only partially reversible.

**Figure 4:**
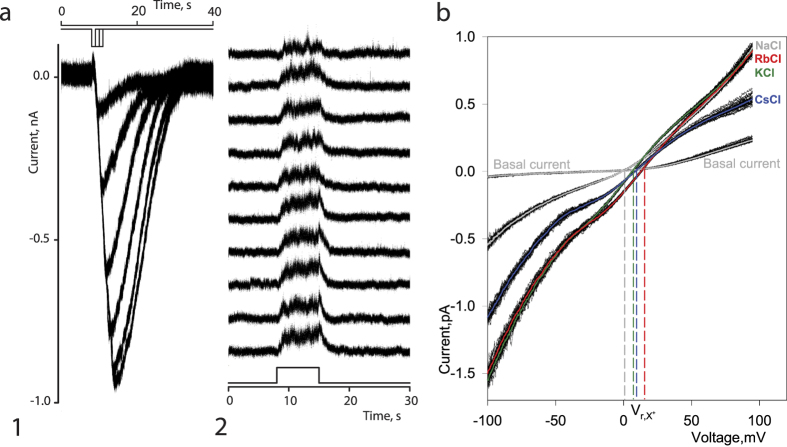
Electrophysiological properties and monovalent cation permeability of CpomOR channels. **(a)** CpomOrco + OR1 expressing HEK cells respond to the unspecific agonist VUAA3 200 μM generating inward currents in dose dependent manner (a1). Experimental conditions: whole-cell voltage clamp recording; holding potential: −50 mV; intracellular solution (mM): NaCl 140, EGTA 0.5, Hepes 10, pH 7.4; extracellular solution (mM): 140 NaCl, 2.0 CaCl_2_, 10 HEPES, pH 7.5. Stimulus intensity was changed by changing stimulus pulse duration. Basal current level was subtracted. VUAA3, 200 μM, applied repeatedly to the extracellular surface of membrane patch in outside-out configuration reversibly increased membrane current noise that can be associated with the activity of ion channels (a2). Experimental conditions: outside-out patch recording; holding potential +50 mV; intracellular solution (mM): KCl 140, EGTA 1, Hepes 10, pH 7.4; extracellular solution (mM): 140 NaCl, 2.0 CaCl_2_, 10 HEPES, pH 7.5; stimulus: VUAA3, 200 μM. Diagrams in a1 and a2 depict a time course of stimulus presentation. Note: the VUAA3 activated OR channels demonstrate little if any rundown. **(b)** To estimate the selectivity of the OR channels to monovalent cations we used whole-cell recordings. VUAA3 (200 μM) was added to all extracellular test solutions. Cells were first exposed to NaCl 140 mM solution. Then, the same whole-cell preparation was exposed to a solution in which the Na^+^ ions were replaced by one of the following cations: Li^+^, K^+^, Cs^+^, Rb^+^. A series of ramps (50–100, black lines) were used for every solution tested and averaged (color lines). A potential at which current voltage characteristic of VUAA3 activated integral current intercepts current voltage characteristic of basal current was used as a Vr of the OR channel current in a given ion conditions (vertical colour lines). To determine the reversal potential shift (ΔVr), the Vr of the currents obtained in symmetrical Na^+^ conditions (VrNa^+^) was subtracted from the Vr obtained, then Na^+^ was replaced by respective cations (VrX). The ΔVrX means were then used to estimate the permeability ratios (PX^+^/PNa^+^). The permeability ratio sequence for some inorganic monovalent cations (PX^+^/PNa^+^) was: Rb^+^ (2 ± 0.12) > K^+^ (1.37 ± 0.03) ≥ Cs^+^ (1.36 ± 0.03) ~ Na^+^ > Li^+^ (0.93 ± 0.06). Experimental conditions: whole-cell voltage clamp recording; holding potential: −60 mV; intracellular solution (mM): NaCl 140, EGTA 0.5, Hepes 10, pH 7.4; extracellular solution: varied in accordance with the above description.

We next used whole-cell and outside-out patch-clamp recordings to characterize the basic electrophysiological properties of the CpomOrco + OR1 complex. All cells expressing CpomOrco + OR1 responded to VUAA3 (200 μM), generating an inward current that varied in amplitude from −49 to −1165 pA, with a mean amplitude of −431 ± 54 pA, n = 37. Un-transfected cells were not sensitive to VUAA3 (12 cells tested). The whole-cell currents gradually increased in a stimulus intensity dependent manner (Fig. 4). In all cases, when cells were stimulated multiple times, the responses were characterized by constant amplitudes and stable kinetic parameters, indicative of the ionotropic nature of the receptors under the current experimental conditions. The results of subsequent experiments using outside-out patch-clamp recordings further support these observations (Fig. 4). VUAA3 (200 μM) applied repeatedly to the extracellular surface of membrane patch reversibly increased the membrane current noise likely associated with the activity of ion channels. As found for the whole-cell currents, the VUAA3 activated ion channel noise of membrane patches demonstrated little if any rundown (Fig. 4). Note, the low single channel conductance and fast gating likely make the unitary currents undistinguishable.

Whole-cell recordings were also performed to estimate the selectivity of the Orco-based channel to a selection of inorganic monovalent cations (Fig. 4; see Methods for details). The permeability ratio sequence for the inorganic monovalent cations tested was (PX^+^/PNa^+^):


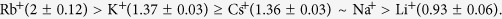


The sequence is consistent with the selectivity sequences previously reported for Orco-based channels from other insects^31^,^32^.

### Deorphanization of codling moth putative PRs

**Figure 5:**
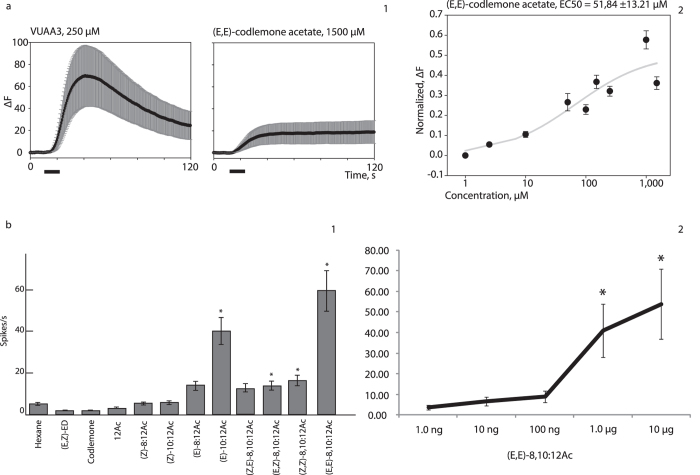
Functional expression of CpomOR6a. **(a)** Functional expression of CpomOrco + OR6a in HEK293T cells. (a1) Amplitudes of the calcium responses (mean of the maximum response ± SEM) to 250 μM VUAA3 positive control (69.71 ± 27.29, dF; left) and to 1500 μM (E,E)-codlemone acetate (18.91 ± 10.31, dF; right); n = 68. Black bar: stimulus. (a2) Normalized dose-response plot to codlemone acetate. **(b)** Functional expression of CpomOR6a in *Drosophila* at1 OSNs. (b1) Mean ± SEM response of CpomOR6a-expressing OSNs stimulated with 10 μg doses of different compounds (Supplementary Dataset S2; n = 10). Asterisks indicate significant differences between the solvent and the indicated compound (Mann-Whitney U Test, *p* < 0.05, n = 9). Spike trains for compounds giving significant difference with the solvent are reported in Supplementary Fig. S5. (b2) Spiking activity of OSNs in response to different doses of (E,E)-codlemone acetate. Asterisks denote significant differences between the solvent and the dose indicated (One-way ANOVA with repeated measures, LSD post-doc test, *p* < 0.05, n = 10).

As the physiological and pharmacological tests demonstrated that both the homomeric and heteromeric PR and Orco complexes were functionally expressed in the HEK293T cell system, we next used the system to look for natural ligands for the receptors. A library of potential codling moth pheromones and synergist compounds (Table 1), as well as additional compounds, such as plant volatiles and volatiles from fermentation and commercial substances (Supplementary Table S1) were screened against CpomOrco + OR6a, CpomOrco + OR3 and CpomOrco + OR1. Treatment with VUAA3 (250 μM) was used as a positive control. In tests with CpomOrco + OR6a, we observed clear activation in response to stimulation with (E,E)-8,10-dodecadien-1-yl acetate ((E,E)-codlemone acetate; Fig. 5). Subsequent experiments were used to calculate an EC50 of 51.84 ± 13.21 μM for codlemone acetate (Fig. 5); however, the amplitude at saturating concentrations (18.91 ± 10.31, dF) was ~27% of the positive control amplitude (69.71 ± 27.29, dF; Fig. 5). Interestingly, compared to the positive control, we observed a long lasting codlemone acetate activation of transfected HEK293T cells, which led to a delayed recovery after stimulation (Fig. 5).

Once we determined the response of CpomOR6a towards codlemone acetate using the HEK293T cell system, in parallel series of experiments we tested activation of CpomOR6a expressed in *Drosophila* at1 OSNs to a new panel of ligands, including codlemone, pear ester, codlemone acetate and structurally related compounds (Supplementary Dataset S2). As in the HEK293T cell system, the strongest response significantly different from the solvent was elicited by (E,E)-codlemone acetate. However, the receptor was also significantly activated by the isomers (E,Z)-8,10-dodecadien-1-yl acetate and (Z,Z)-8,10-dodecadien-1-yl acetate along with (E)-10-12-dodecadien-1-yl acetate (Fig. 5 and Supplementary Fig. S5). Furthermore, dose response experiments with CpomOR6a towards (E,E)-codlemone acetate demonstrated that the threshold for detection was 1.0 microgram (Fig. 5).

**Figure 6:**
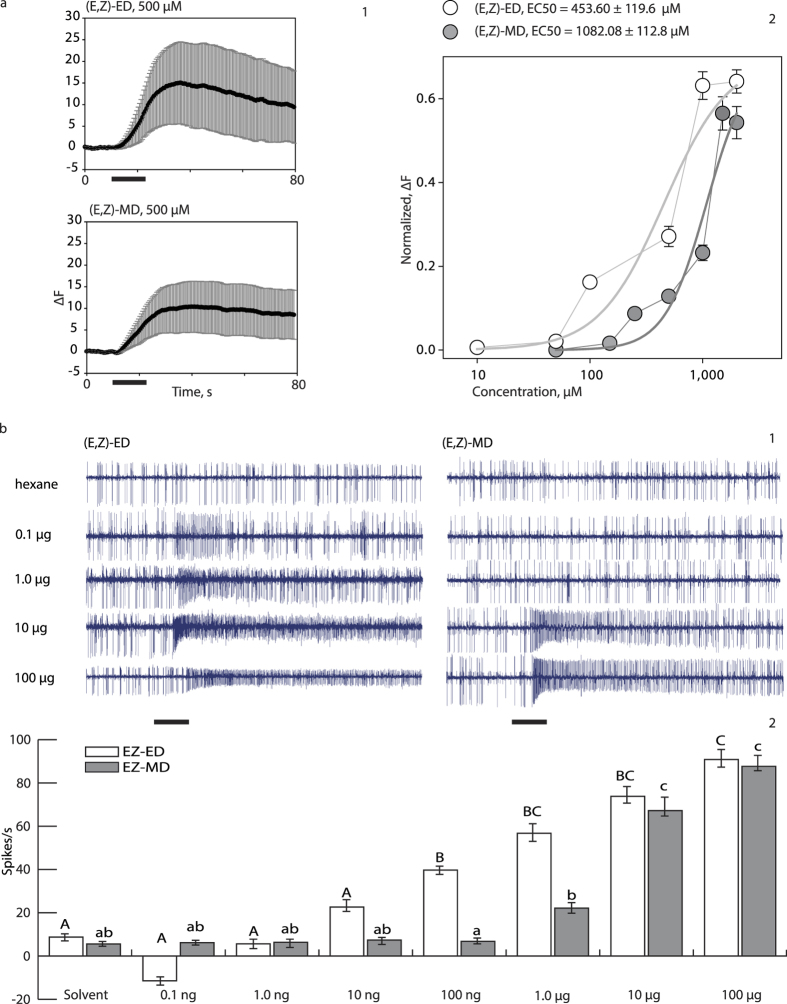
Functional expression of CpomOR3. **(a)** Functional expression of CpomOrco + OR3 in HEK293T cells. (a1) Comparison of CpomOrco + OR3 amplitudes of the calcium responses (mean of the maximum response ± SEM) to 500 μM pear ester (15.07 ± 9.48, dF; left) and to 500 μM methyl ester (10.40 ± 5.91, dF; right); n = 151. Black bar: stimulus. (a2) Normalized dose-response of pear ester [(E,Z)-ED, white] and methyl ester [(E,Z)-MD, grey]. **(b)** Functional expression of CpomOR3 in *Drosophila* ab3A OSNs. (b1) Spiking activity of OSNs in response to different doses of (E,Z)-ED (left) and (E,Z)-MD (right). Black bar: stimulus (500 ms). (b2) Mean ± SEM response of CpomOR3-expressing OSNs stimulated with different doses of (E,Z)-ED (white, n = 13) and (E,Z)-MD (grey, n = 13). Repeated measures ANOVA determined that different doses of the compound elicited significant differences (F(7, 91) = 42.17, *p* < 0.001). Post hoc tests using the Bonferroni correction revealed that CpomOR3 needed a minimum dose of 100 ng of (E,Z)-ED to elicit a response significantly different from the solvent (*p* = 0.026). On the other hand, for the dose response of (E,Z)-MD a repeated measures ANOVA determined that different doses of (E,Z)-MD also elicited significant differences in CpomOR3 (F(7, 84) = 41.68, *p* < 0.001). Post hoc tests using the Bonferroni correction revealed that OR3 needed a minimum dose of 10 μg of (E,Z)-MD to elicit a response significantly different from the solvent (*p* = 0.020).

Previously we demonstrated that CpomOR3 expressed in ab3A and at1 OSNs is sensitive to pear ester^25^, and here we confirmed this result using the HEK293T cell expression system: pear ester [(E,Z)-ED] elicited a response from cells expressing CpomOrco + OR3 (Fig. 6). Furthermore, we found that an analogous ester emitted by pear (methyl (E,Z)-2,4-decadienoate [(E,Z)-MD])^33^, also activates CpomOrco + OR3. EC50 estimations (EC50_HEK-(E,Z)-ED_ = 453.60 ± 119.6 μM; EC50_HEK-(E,Z)-MD_ = 1082.08 ± 112.8 μM) and dose-response plots (Fig. 6) suggest that CpomOrco + OR3 has a lower specificity for (E,Z)-MD than for (E,Z)-ED. As with CpomOrco + OR6a responses to (E,E)-codlemone acetate, we observed a slow recovery after CpomOrco + OR3 stimulation with (E,Z)-MD (Fig. 6).

For the dose response of CpomOR3 when expressed in *Drosophila* ab3A OSNs (Fig. 6), a minimum dose of 100 ng loaded in the stimulus cartridge was required to elicit a response significantly different from the solvent for (E,Z)-ED and of 10 μg for (E,Z)-MD (Fig. 6). Application of a different heterologous expression system confirmed CpomOR3 sensitivity to both pear ester and its analogous (E,Z)-MD, with further suggesting lower specificity for (E,Z)-MD than (E,Z)-ED.

In contrast to CpomOR3 and CpomOR6a, we were unable to identify any ligands that activated CpomOrco + OR1. While calcium imaging experiments with VUAA1 and VUAA3 suggest that CpomOR1 is likely functionally expressed in the HEK293T system (Fig. 2), the receptor failed to respond to any of the ligands tested. In agreement with these results, CpomOR1 also failed to respond in *Drosophila* at1 OSNs to pheromones and synergists (spikes/s = 0.61, n = 5; response minus basal activity, see methods section for detail) or their combinations with codlemone (spikes/s = 0.00, n = 5; Supplementary Dataset S3).

## Discussion

Of the 58 ORs identified in *C. pomonella*^11^, 12 belong to the PR clade. Here we functionally expressed three of them, CpomOR1, CpomOR3 and CpomOR6a, in an HEK293T expression system with the goal of identifying their natural ligands. We found that in contrast to early studies that demonstrated that a channel forming subunit Orco (OR83b) is required as a chaperon to target the ligand-specific insect OR subunits to plasma membrane^5^, CpomORs are properly expressed and targeted when independently expressed in HEK293T cells (Fig. 1). Like all functionally characterized insect ORs, the codling moth receptors described here can be activated by a group of common synthetic agonists including the VUAA compounds^28^ that interact directly with Orco, indicating that they are indeed functional in HEK293T cells. Two criteria based on calcium responses to VUAAs can be used to functionally confirm the expression of the orphan ORs: (1) heteromeric OR complexes are more sensitive to the agonists as compared to homomeric Orcos (Fig. 2) and (2) the parameters of the calcium responses mediated by the activity of heteromeric CpomOrco + OR complexes are characterized by faster activation/deactivation kinetics (Fig. 2). These observations are consistent with data for heterologously expressed mosquito ORs^28^,^34^ and may suggest different functional modes of homomeric and heteromeric complexes. Further studies are necessary to understand the mechanisms underlying these differences.

One of the emerging characteristic pharmacological properties of insect OR-based channels is sensitivity to ADs^29^,^30^. Extracellular application of the AD MIA almost completely blocked the VUAA3-activated calcium signal. Similar results were observed of both homomeric (CpomOrco) and heteromeric CpomOrco + OR complexes. These results are consistent with the idea that all insect or, perhaps, even all arthropod chemosensory receptor channels (among ORs and IRs) can be characterized by somewhat common pharmacology^29^,^30^,^35^,^36^,^37^. Despite their apparent lack of selectivity, some ADs tested have demonstrated different antagonistic potencies against different OR complexes^29^, suggesting that broader screening of AD library may identify compounds with greater OR channel selective affinity.

Among the three CpomORs tested, CpomOR6a and CpomOR1 in particular were found to be robustly expressed in male moths^10^,^11^ and were thus initially proposed to be candidate receptors for codlemone. While we did not observe any response to codlemone with either (Supplementary Dataset S2, S3 and Supplementary Fig. S4), we did find that CpomOR6a interacts with codlemone acetate and its isomers (Fig. 5), pheromone components found in both *C. pomonella* and related species^22^,^38^,^39^. The lower amplitude of the CpomOrco + OR6a responses to codlemone acetates may suggest that these compounds are partial agonists of the receptor. Another possible explanation is that only approximately 30% of receptors expressed represent heteromeric complexes sensitive to their cognate ligand/s with the majority of the receptors being homomeric (sensitive exclusivily to common agonists, VUAAs). It remains necessary to determine whether a different ratio of OR/Orco expression would yield a higher ratio of heteromeric/homomeric OR complexes and thus different amplitudes in the response.

All four geometric isomers of codlemone acetate are reported to be pheromone compounds in tortricid species. While codlemone acetate is a minor pheromone component for *C. pomonella* that acts as a subtle behavioural synergist when blended at a low level with the main codling moth sex pheromone codlemone, it is a major pheromone component in other species and higher concentrations have a strong antagonistic effect on codling moth attraction^26^. Species closely related to *C. pomonella*, like *C. nigricana, C. splendana, C. pyrivora*^22^*, C. latiferreana*^40^,^41^ *and Hedya nubiferana*^38^,^39^, use codlemone acetate as their main pheromone component. While speculative, a possible explanation of the existence of the codlemone acetate receptor in *C. pomonella* may be as a remnant of the former ancestor of the insect. However, conserving a receptor dedicated to detection other species may be important for reproductive isolation. Otherwise, since the pheromone is also emitted by moths within the same host range, their detection may facilitate host finding for *C. pomonella*. The arise of a receptor specialized for the detection of a main pheromone compound like codlemone, may likely represent a step towards allopatric speciation of the codling moth.

In *C. pomonella*, codlemone acetate isomers are detected by two types of OSNs located in sensilla trichodea on male antennae^42^, one of which responding primarily to the main geometric isomer of codlemone [(E,E)] with tenfold less sensitivity to other geometric isomers [(Z,E); (E,Z); (Z,Z)]. These OSNs are even less responsive to (E,E)-codlemone acetate and its geometric isomers [(Z,E); (E,Z); (Z,Z)]. The second type of OSNs detects all geometric isomers of codlemone acetate, with the (E,E)-isomer eliciting the strongest response, but is insensitive to all geometric isomers of codlemone. Our structure-activity SSR recordings (Fig. 5b) indicate that apart from (E,E)-codlemone acetate, CpomOR6a is able to detect the isomers (E,Z)- and (Z,Z)-codlemone acetate, and possibly also (Z,E)-codlemone acetate, along with (E)-10:dodecadien-1-yl acetate (Supplementary Fig. S5), which correlates with the electrophysiological results obtained by Bäckman *et al*. (2000)^42^. Considering that this receptor does not respond towards codlemone, there must be another PR expressed by the type of OSNs that responds primarily to codlemone but also weakly to codlemone acetate. Walker *et al*. (2016)^11^ have proposed CpomOR1 as the most likely candidate for codlemone detection based on its transcript abundance. Although we were unable to demonstrate its responsiveness with both expression systems, future deorphanization attempts will unveil if this prediction holds true. Another remaining question is whether the transcript variant OR6b^11^ have the same response spectra as OR6a and what might be its relevance, especially considering the lack of knowledge in alternative splicing in lepidopteran PRs^43^.

CpomOR3 is sensitive to pear ester [(E,Z)-ED], a non-pheromone compound, when expressed in *Drosophila* at1 and ab3A OSNs^25^. Heterologous expression of CpomOR3 in the HEK293T system confirmed this finding and allowed identification of another potential ligand, the analogous methyl-(E,Z)-2,4-decadienoate [(E,Z)-MD] (Fig. 6). As found when it was expressed in ab3 sensilla (Fig. 6), CpomOR3 was more sensitive to (E,Z)-ED than to (E,Z)-MD. The difference between these two compounds, one carbon of the alkyl group, may determine different binding affinity, perhaps due to differences in the polarity of the compounds. This is consistent with our previous observations of different sensitivities reported for CpomOR19 when expressed in ab3 basiconic sensilla responding to different types of alkyl-1-indanones^44^. Recent studies suggest that the blend of codlemone and (E,Z)-ED produce strong synergistic effect on the activity of adjacent glomeruli specifically tuned to (E,Z)-ED and codlemone^9^,^24^. These results indicate that while it may be at least partially responsible for the synergistic effects of pheromones and pear ester, CpomOR3 may not be a pheromone receptor, per se. Interaction of (E,Z)-MD with the same receptor that recognizes (E,Z)-ED, may result in a similar effect at the neural network level when combined with codlemone, but this remains to be tested.

The identification of a potential PR agonist is one of the first successful associations between an insect PR and a likely pheromone ligand using HEK293T-based heterologous expression^45^,^46^,^47^. Among insect ORs, PRs have been especially difficult to functionally characterize^48^,^49^, as illustrated even by our own experiments, where CpomOR1 does not produce any response to pheromones, synergists and their combination (Table 1 and Supplementary Dataset S3). These results highlight the importance of developing alternative testing methods. Overall, our findings suggest that the potential codling moth pheromone receptors exhibit biophysical and pharmacological properties surprisingly similar to insect ORs when expressed heterologously: CpomOrco-CpomOR complexes are capable of functioning as ionotropic receptor channels and can be characterized by relatively low conductance and fast gating parameters; CpomOrco-CpomOR complexes are activated by the group of common compounds, VUAAs, and can be efficiently blocked by amiloride derivatives. Several ligands were identified: codlemone acetates, pear ester and the analogous methyl ester. These findings provide important insights into our understanding the mechanisms of the moth chemoreceptor functioning and, consequently, for developing pest control strategies based on disruption of pest chemosensory communication.

## Methods

### Insect dissection and RNA extraction

*C. pomonella* pupae were obtained from a laboratory rearing (Andermatt Biocontrol, Grossdietwil, Switzerland), and adults were allowed to emerge in cages kept at 23 °C, 70 ± 5% RH and 16 h : 8 h light/dark cycle, and were fed 10% sugar solution. For dissections, 2–3 day old females and males were used. Using sharp forceps, antennae were removed at the base of the pedicel and immediately flash-frozen using liquid nitrogen, and thereafter kept at −80 °C. RNA was extracted using the RNeasy kit (Qiagen, Hilden, Germany), which included a DNase digestion to eliminate genomic DNA contamination. Antennal RNA was quantified using Nanodrop (8000 UV-vis Spectrophotometer, Thermo Scientific, Wilmington, DE, USA).

### Rapid amplification of cDNA ends (RACE) PCR

While the full length sequences of *CpomOrco, CpomOR3*, and *CpomOR1* were previously reported^10^,^25^, RACE PCR was performed to obtain the complete open reading frame of *CpomOR6a*. cDNA was reverse-transcripted from antennal RNA using the SMARTer kit (Clontech, Mountain View, CA, USA). Primer sequences were designed using existing contig data as reference, and thermodynamical features were checked by OligoEvaluator (Sigma Genosys, http://www.oligoevaluator.com/). Putative oligodimerization was checked by OligoAnalyzer 3.1 (Integrated DNA Technologies, http://eu.idtdna.com/calc/analyzer), and melting temperatures were estimated using the salt-adjusted algorithm of the OligoCalc website (http://www.basic.northwestern.edu/biotools/OligoCalc.html). For primers, the goal was a GC% 40–60, Tm < 70 °C, and to create a product with at least 150 bases of overlap with existing contig data. The designed sequence of the 5′_OR6a primer, which successfully extended the CDS of *CpomOR6a*, is reported in Table 2. SMARTer RACE PCR was performed using an adjusted version of the supplied protocol. Supplied thermostable DNA polymerase was used with a temperature program of 95 °C for 2 min, followed by 30 cycles of 95 °C for 1 min, 65.42 °C for 90 s 68 °C for 2 min, and a final elongation of 68 °C for 7 min. The 5′_OR6a primer was combined together with Universal primer A mix supplied in the kit, with 2% DMSO per reaction volume added. PCR products were analysed by electrophoresis on 1.5% agarose gel. Bands were visualized after staining with ethidium bromide using a Gel Doc XR (Bio-Rad, Hercules, CA, USA). Relevant bands were excised and purified by the Gel extraction kit (Qiagen). Quantification was performed using a Nanodrop 3300 Fluorospectrometer (Thermo Scientific) using the PicoGreen^®^ dsDNA reagent kit (Molecular Probes, Life Technologies).

Samples were sequenced (Sanger sequencer, 3730xl Applied Biosystems, Life Technologies) using gene specific primers. The 5′ sequenced region was assembled with existing contig data and the candidate CDS was identified using the online tool ORF Finder (http://www.ncbi.nlm.nih.gov/gorf/orfig.cgi).

For functional expression, total RNA extracted from male and female antennae were submitted to full-length cDNAs synthesis using RT-for-PCR kit (Clontech), and the full length CDS was amplified (primers Fw_OR6a and Rv_OR6a in Table 2) and sequenced to confirm that the assembly was correct. The nucleotide sequence was converted to amino acids using the ExPASy translate tool (http://web.expasy.org/translate/), after which transmembrane domains were predicted using TMHMM v2.0 (http://www.cbs.dtu.dk/services/TMHMM/) and TOPCONS (http://topcons.cbr.su.se/)^50^. The Topology of the transmembrane protein was visualized using TOPO 2.0 (http://www.sacs.ucsf.edu/cgi-bin/open-topo2.py). The completed sequence of OR6a has been updated in Genbank, JN836671^11^.

### Cloning of olfactory receptors for heterologous expression in HEK293T cells

In order to produce amplicons suitable for cloning into pDONR221 (Invitrogen Life technologies, Grand Island, NY, USA), we inserted *att*B regions suitable for BP-clonase-recombination upstream of the CDS primer sequences (*att*B1 forward region: 5′-GGGGACAAGTTTGTACAAAAAAGCAGGCTTAACA-3′; *att*B2 reverse region: 5′-GGGGACCACTTTGTACAAGAAAGCTGGGT-3′, Gateway Technology, Invitrogen). CDS primers (Table 2) were designed to amplify full-length CpomOR sequences (Genbank database accession numbers, CpomOrco: JN836672; CpomOR6a: JN836671; CpomOR3: KJ420588; CpomOR1: JN836674.1). For forward primers, a *NotI* restriction site (5′-GCGGCCGC-3′) followed by the HEK-cell optimized 5′-CACC-3′ Kozak sequence (Dr. Jacob Corcoran, personal communication) and the gene-specific forward sequence, were located downstream of the *att*B1 sequence. For reverse primers, an *ApaI* restriction site (5′-GGGCCC-3′) followed by the reversed-stop codon (5′-TTA-3′) and the gene-specific reverse sequence, were located downstream of the *att*B2 sequence. To create V5-N-terminal variants suitable for immunohistochemical experiments, 42 nucleotides (5′-GGCAAGCCTATCCCTAATCCTCTGCTGGGCCTGGACAGCACC-3′) coding for 14 additional amino acids of a V5-epitope tag (Nt-GKPIPNPLLGLDST-Ct) were added to the forward primer between the start codon and the rest of the gene-specific forward sequence.

Amplification was performed with Advantage 2 polymerase (Clontech) using a temperature program of 94 °C for 5 min followed by 35 cycles of 94 °C for 1 min, Tm of the primer for 1 min and 68 °C for 2 min with a final elongation step of 68 °C for 7 min. A 4.0 μL PCR volume was mixed with 1.0 μL of BP-clonase (Gateway Technology, Invitrogen) and 150 ng of pDONR221 (Invitrogen), and was incubated for 4 h at 25 °C. Of this reaction volume, 2.0 μL was used to transform TOP10 competent cells (Invitrogen). After transformation, 50 μL of the reaction was plated on 50 μg/mL Kanamycin selective media and incubated overnight at 37 °C.

Colonies were sampled, and diluted in 50 μL selective LB media with 50 μg/mL Kanamycin, to be grown for 2 hours at 37 °C and 225 rpm. Colony PCR was performed to confirm inserts, using 1.0 μL culture from single colony-volumes with the M13FW universal primer and the relevant reverse OR-primer. Amplifications were performed using the GoTaq Green Master Mix (Promega, Fitchburg, WI, USA) with a temperature program of 95 °C for 15 min, followed by 35 cycles of 95 °C for 45 s, 55 °C for 1 min, 72 °C for 2 min, and a final elongation of 72 °C for 7 min. Colony PCR samples were analysed as described above. Cultures producing relevant bands in colony PCR were grown at 37 °C and 225 rpm overnight in 5.0 mL selective LB media with 50 μg/mL Kanamycin. The pDONR221 plasmids containing CpomOR ORFs were purified using a miniprep kit (Qiagen). Plasmid quantification was performed using Nanodrop (8000 UV-vis Spectrophotometer), and samples were sequenced (Sanger sequencer, 3730xl) using M13 universal primers.

A 2.0 μg aliquot of each pDONR221/CpomOR and pDONR221/V5-CpomOR DNA was digested overnight at the limit of star activity, in a reaction volume with 0.5X FastDigest *NotI* and *ApaI* added (Thermo Scientific), following the recommended protocol. Reaction volumes were run on 1.5% agarose gel and visualized after staining with ethidium bromide and digested bands were purified by Gel extraction kit (Qiagen). Quantification was performed as described above. From the purified bands, 50 ng of the reaction was combined with 50 ng pcDNA5/TO (Invitrogen Life technologies) previously digested and purified, 1.0 U T4 DNA ligase and 1X of the supplied reaction buffer (Thermo Scientific), which was incubated 2 h at room temperature for ligation. Of this reaction volume, 2.0 μL was used to transform TOP10 competent cells. Colony PCR was performed to screen positive colonies, and colonies selected for correct inserts were amplified, vectors were extracted and purified by miniprep, and sequenced (Sanger sequencer, 3730xl). In order to perform heterologous expression, pcDNA5/TO/CpomORs and pcDNA5/TO/V5-CpomORs were scaled up using GeneJet Plasmid Midiprep Kit (Qiagen).

### Heterologous expression in HEK293T cells and transient transfection

HEK293T cells were grown in HEK cell media [Dulbecco’s modified Eagle’s medium containing 10% fetal bovine serum (MP Biomedicals, Solon, OH, USA), 2.0 mM L-glutamine, and 100 μg/mL Penicillin/Streptomycin (Invitrogen)] at 37 °C and 5% CO_2_. To test transient expression of CpomORs for calcium imaging or patch-clamp recording experiments, 35-mm petri dishes containing semi-confluent HEK293T cells were transiently transfected. To transfect cells with CpomOrco, we used 1.0 μg of pcDNA5/TO/CpomOrco DNA. In order to promote expression of the OR^51^, we used double aliquots for pcDNA5/TO/CpomOR DNAs (2.0 μg), combined with 1.0 μg of pcDNA5/TO/CpomOrco for co-transfections (CpomOrco + ORs). To report expression for calcium imaging experiments, 1.0 μg of a separate plasmid DNA [pEBFP2-Nuc, a gift from Robert Campbell (Addgene plasmid # 14893^52^)] carrying the coding sequence for a blue fluorescent protein (BFP) was used. In patch-clamp recordings, 1.0 μg of a separate plasmid DNA (pXOOM, Clontech) carrying the coding sequence for a green fluorescent protein (GFP) was used to report expression. In order to report candidate OR-expressing cells, expression of both fluorescent reporter genes was under the regulation of the same promoter for CpomOR genes (CMV). Transfection DNAs were dissolved in 100 μL sterile DMEM, mixed with 3.0 μL Calfectin (SignaGen, Rockville, MD, USA) following the recommended protocol. To estimate transfection efficiency, a parallel transfection was conducted using the positive control vector pcDNA5/TO/LACZ (Invitrogen) and staining with 0.1% XGal^53^. The predominant majority of LACZ transfected cells demonstrated a strong staining indicating a high transfection efficiency.

Transfections were conducted overnight. HEK cell media was replaced with 1.0 mL fresh media to incubate cells at 37 °C for up to 6 h, at which point part of the cell culture was spread in the middle of a 35-mm plate as individual cells or small clusters. After 12 h of incubation at 37 °C and 5% CO_2_, cells were rinsed at the sides with 2.0 mL fresh HEK media. Cells were allowed to recover for at least 1 h prior to calcium imaging.

### Immunohistochemistry

To study membrane localization of ORs in HEK293T, cells were transfected with pcDNA5/TO/V5-CpomOrco or co-transfected with pcDNA5/TO/CpomOrco combined with pcDNA5/TO/V5-CpomORs. To compare heterologous expression of ORs alone, further transfections were prepared for pcDNA5/TO/V5-CpomORs without CpomOrco DNA. V5-CpomOrco was used as a positive control. Non-transfected HEK cells were used as a negative control. After growth, cells were split into 12-well plates, each containing a single 12 mm cover slip, previously sterilized with ethanol and 10 min UV-light/side, and coated with matrigel matrix (Corning, Tewksbury, MA, USA) diluted 1:40 in DMEM. After overnight growth at 37 °C and 5% CO_2_, cover slips were washed gently with room temperature Hank’s Balanced Salt Solution 1X (HBSS, Invitrogen) and incubated 15 minutes on ice soaked in ice-cold 100% methanol (Sigma Aldrich, St. Louis, MO, USA). After incubation, methanol was removed, and cover slips were washed twice with HBSS 1X and stained overnight at 4 °C with V5 Epitope Tag Antibody, DyLight 488 conjugate (E10/V4RR) (Thermo Fisher Scientific, Waltham, MS, USA) diluted 1:100 in staining solution^54^. The cover slips were washed twice with HBSS 1X, and placed on microscope slides (Fisher Scientific, Pittsburg, PA, USA) with one drop of DAPI-fluoromont-G (Southern Biotech, Birmingham, AL, USA), for analysis by confocal microscopy.

### Confocal microscopy

Samples were analysed with Leica TCS-SP5 Confocal Laser Scanning Microscope (Leica Microsystems, Wetzlar, Germany) using HCX PL APO CS 63.0 × 1.20 WATER UV lens, 1.33 refraction index. Scanner settings were calibrated with PinHole (m): 133.6 μm; PinHole (airy): 1.2; Zoom: 1.7. Images were taken step sizing the size-depth, optimizing the number of section by halving the numbers provided by the system. Hardware was set to have all lasers active (405 Diode, UV; Argon, Visible; DPSS 561, Visible; and HeNe 633, Visible) with Argon, Visible at 29%. In order to distinguish nuclei fluorescence from antibody-labelled plasma membrane extrusions, DAPI was exited using pre-set DAPI parameters, calibrating Laser Line UV (405) at 27% and all other Laser Line at 0%. Emission PMT was calibrated between 417 and 496 nm, Gain: 693 nm, Offset: 0, Transmission: 504, Offset: 0. To detect DyLight Antibody with excitation/emission rate 493/518 nm, pre-set FITC parameters were adopted, calibrating Laser Line visible (488) at 76% and all other Laser Line at 0%. Emission PMT was calibrated between 500 and 560 nm, Gain: 808 nm, Offset: 0, Transmission: inactive. All parameters were adjusted by the company-provided software (Leica Microsystems LAS AF TCS MP5). Images were analysed using the same software and elaborated using ImageJ 1.42 (available from public domain at http://rsbweb.nih.gov/ij/index.html).

### Calcium imaging

To test activation of olfactory receptors, CpomOrco or CpomOrco + ORs HEK293T cells were incubated for 1 h at room temperature in 0.5–1.0 mL HEK cell Ringer (mM: 140 NaCl, 5.0 KCl, 1.0 CaCl_2_, 1.0 MgCl_2_, 10 HEPES, 10 Glucose, pH 7.5) containing the fluorescent calcium indicator Fluo-4AM (Invitrogen) at 5–15 μM prepared with 0.2–0.06% Pluronic F-127 (Invitrogen). After incubation, the buffer was removed and cells were rinsed with 4.0 mL fresh HEK Ca^++^ Ringer (mM: 140 NaCl, 2.0 CaCl_2_, 10 HEPES, pH 7.5), and placed on the stage of an inverted microscope (Olympus IX-71, Olympus Corp., Tokyo, Japan) equipped with a cooled CCD camera (ORCA R2, Hamamatsu, Hamamatsu City, Japan). Cells were continuously superfused with Ca^++^ Ringer using two gravity fed perfusion contours. The stimulating contour washing the cells (~250 μL/min) was switched rapidly to the stimulus contour using a multi-channel rapid solution changer (RSC-160, Bio-Logic, Claix, France) under the software control of Clampex 9 (Molecular Devices, Sunnyvale, CA, USA).

Fluorescence imaging was performed using Imaging Workbench 6 software (INDEC BioSystems, Santa Clara, CA, USA). Each cell was assigned a region of interest (ROI) and changes in fluorescence intensity within each ROI were measured and expressed as the fractional change in fluorescence intensity (dF) or in some cases as dF/F0 where F0 is the basal fluorescence level before agonist application. Stored time series image stacks were analysed off-line using Imaging Workbench 6, Clampfit 10.5 (Molecular Devices), SigmaPlot 11 (Systat Software Inc., San Jose, CA, USA) or exported as TIFF files into ImageJ 1.42. Continuous traces of multiple responses were compensated for slow drift of the baseline fluorescence when necessary. All recordings were performed at room temperature (22–25 °C).

### Dose/response characteristics of VUAA-compounds

Non-specific agonists Acetamide,*N*-(4-ethylphenyl)-2-[[4-ethyl-5-(3-pyridinyl)-4*H*-1,2,4-triazol-3-yl]thio]- (**VUAA1**), CAS 525582-84-7 (Glixx Laboratories, Southborough, MA, USA) and Acetamide,2-[[4-ethyl-5-(4-pyridinyl)-4*H*-1,2,4-triazol-3-yl]thio]-*N*-[4-(1-methylethyl)phenyl]- (**VUAA3**), CAS 585550-72-7 (Molport, Riga, Latvia), were dissolved in Dimethyl Sulfoxide (DMSO, Sigma Aldrich) and stored as a stock solutions (100 mM) at −20 °C. The final working concentrations of VUAA1 and VUAA3 were usually prepared right before the experiments. Amplitudes of the calcium responses were used to generate dose-response characteristics. The values were normalized to the response amplitude recorded at 1000 μM VUAA1/VUAA3. The amiloride derivative 5-(N-methyl-N-isobutyl)amiloride (MIA), CAS 2609-46-3 (Sigma Aldrich) was prepared as DMSO stock solution (100 mM) and used to block VUAA3 activated calcium signals at the working concentration 100 μM.

### Screening of ligand candidates

HEK293T cells expressing CpomOrco + OR6a, CpomOrco + OR3 and CpomOrco + OR1 were stimulated with an array of compounds such as: potential insect pheromone compounds and plant volatile synergists active on the olfactory system and behaviour of the codling moth^1^,^25^, non-host plant volatiles active on other tortricids^55^, the methyl pear-ester^33^ (Table 1) and further compounds among plant/fruit volatiles, volatiles from fermentation and commercial substances (Supplementary Table S1).

Physical parameters were obtained from Scifinder (Scifinder, 2015; Chemical Abstracts Service: Columbus, OH, 2015; accessed Jan-Nov 2015) and Chemspider (http://www.chemspider.com/). Compounds were diluted in either DMSO or ethanol (Sigma Aldrich) depending on their solubility properties. Each compound was tested at 100 μM (10 s stimulus duration). VUAA1 100 μM was used as a positive control in all cases (stimulus duration 5.0 s). Responses to codlemone acetate (for CpomOR6a) and pear and methyl esters (for CpomOR3) were normalized to the 250 μM VUAA3 evoked calcium signal response amplitude. Following modification, the Hill equation, (F = F_max_*[A]^h^/([A]_1/2_^h^ + [A]^h^), was used to fit the concentration dependence data for agonist/ligand dependent OR channel activation, where **F**s are the normalized fluorescent intensity values, **[A]** is the agonist/ligand concentration, **[A]**_**1/2**_ is the half-effective concentration (EC50), and **h** is the cooperativity coefficient.

### Electrophysiology and data analysis

GFP positive HEK293T cells were visualized using either an Axiovert 100 inverted microscope (Carl Zeiss, Inc., München, Germany) equipped with a mercury vapour compressed-arc lamp (HBO100) coupled to widefield fluorescence filter set (1114-459, Carl Zeiss, Inc.) or Olympus IX-71 inverted microscope described above (Olympus Corp.). The OR channel activity was investigated using whole-cell and outside-out patch-clamp recordings. The currents were measured with a 200B patch-clamp amplifier (Molecular Devices) and a digital interface (Digidata 1320 A, Molecular Devices), low pass filtered at 5.0 kHz, sampled at 1–2 kHz. Analysis of the data was carried out using pCLAMP 9.2/10.5 software (Molecular Devices) and SigmaPlot 10 (Systat Software Inc). OR channel related currents were investigated at a holding potential of −50/+50 mV unless otherwise specified. The polarity of the currents/voltages was presented relative to intracellular membrane surface. Appropriate corrections for liquid junction potentials were made when necessary. Patch pipettes were fabricated from borosilicate capillary glass (BF150-86-10, Sutter Instrument, CA, USA) using a Flaming-Brown micropipette puller (P-87, Sutter Instrument). Only patches with initial cell-attached seal resistance estimated higher than 1.0 GOhms were used in the experiments. Intracellular (pipette) solution for whole-cell experiments was NaCl 140 mM, EGTA 0.5 mM, Hepes 10 mM, pH 7.4 (adjusted with Tris-base, standard Na^+^ 140 mM), while bath solution was usually NaCl 140 mM, CaCl_2_ 1.0 mM, MgCl_2_ 0–1.0 mM, KCl 5.0 mM, Hepes 10 mM, pH 7.4 (adjusted with Tris-base or NaOH). The OR channel current noise was recorded using outside-out patches excised from HEK293T cells and evoked by stimulation with 250 μM VUAA3. The electrode solution was KCl 140 mM, EGTA 2.0 mM, Hepes 10 mM, pH 7.4, while bath solution was NaCl 140 mM, EGTA 2.0 mM, Hepes 10 mM, pH 7.4. Whole-cell recordings were used to estimate the selectivity of the OR channel to monovalent cations. The intracellular solution was standard Na^+^ 140 mM solution. VUAA3 (200 μM) was added to all extracellular test solutions. Cells were first exposed to standard NaCl 140 mM solution. Then, the same cell was exposed to a solution (LiCl 140 mM; KCl 140 mM; CsCl 140 mM; RbCl 140 mM) in which the Na^+^ ions were replaced by one of the following cations: Li^+^, K^+^, Cs^+^, Rb^+^. The whole-cell current-voltage characteristics were generated using series of 15-ms step at -100-(-110) mV followed by a 150-ms voltage ramp (linear change in voltage ~0.67 mV/ms) from -100-(-110) mV to +90–100 mV were applied from a holding potential of -50-(−60) mV. The interval between sweep starts was 1.0 s. Series of current traces (50–100 whole-cell I-V curves) were then averaged and a potential at which current voltage characteristic of VUAA3 activated integral current intercepts current voltage characteristic of basal current was used as a reversal potential (Vr) of the OR channel current in a given ion conditions. To determine the reversal potential shift (ΔVr), the Vr of the currents obtained in symmetrical Na^+^ conditions (VrNa^+^) was subtracted from the Vr obtained, then Na^+^ was replaced by respective cations (VrX). The ΔVrX means were then used to estimate the permeability ratios (PX^+^/PNa^+^) using the Goldman-Hodgkin-Katz potential equation (E_r,__X_ − E_r,__Na_ = (RT/F)ln(P_Na_[Na^+^]_o_/P_X_[X^+^]_i_)^56^.

### Heterologous expression of CpomORs in *D. melanogaster* OSNs

To perform heterologous expression in *D. melanogaster* OSNs, CpomORs were amplified using primers in Table 2 and purified PCR products were cloned into the PCR8/GW/TOPO plasmid (Invitrogen) following the recommended protocol. Cassettes with inserts were then transferred from their PCR8/GW/TOPO plasmids to the destination vector (pUASg-HA.attB, constructed by E. Furger and J. Bischof, kindly provided by the Basler group, Zürich), using the Gateway LR Clonase II kit (Invitrogen). The integrity and orientation of inserts was confirmed by sequencing (Sanger sequencer, 3730xl). Transformant *UAS-CpomOR3* and *UAS-CpomOR1* lines were generated by Best Gene (Chino Hills, CA, USA), using the PhiC31 integrase system, while the transformant *UAS-CpomOR6a* line was generated in our labs. Briefly, recombinant pUASg-HA.attB-CpomOR plasmids were injected into embryos of a *D. melanogaster* line containing an *attP* insertion site within the third chromosome (genotype y1 M{vas-int.Dm}ZH-2A w*; M{3xP3-RFP.attP}ZH-86Fb), leading to non-random integration. To drive expression of *CpomORs* in at1 OSNs in place of the endogenous receptor Or67d, transformant *UAS-CpomOR* lines were crossed to the knock-in *Or67d*^GAL4^ line (kindly provided by Barry Dickson) to generate double homozygous lines *w*^+^;*UAS-CpomOR*; *Or67d*^GAL4^. Additionally, to drive expression of CpomOR3 in ab3A OSNs, the *UAS-CpomOR3* line was crossed to the *Δhalo*;*Or22a-Gal4* mutant line^20^,^57^. To verify insertion of *UAS-CpomORs* constructs into the genome, gDNA was extracted and used as template in PCR with primers for the full ORF of *CpomORs* (Table 2).

### Single sensillum recordings

CpomOR1 and CpomOR6a expressed in at1 OSNs, and CpomOR3 expressed in ab3A OSNs, were tested through single sensillum recordings (SSR). Three to 8-day-old flies were immobilized in 100 μL pipette tips with only the top half of the head protruding. The left antenna of each insect was gently pushed with a glass capillary against a double-sided adhesive tape placed on a piece of glass. This piece of glass and the pipette tip were fixed with dental wax on a microscope slide. Electrolytically sharpened tungsten electrodes (Harvard Apparatus Ltd, Edenbridge, United Kingdom) were used to penetrate the insect´s body. The reference electrode was manually inserted in the right eye of the fly, while the recording electrode was manoeuvred with a DC-3K micromanipulator equipped with a PM-10 piezo translator (Märzhäuser Wetzler GmbH, Wetzler, Germany) and inserted at the base of the determined sensilla. Signals coming from the olfactory sensory neurons were amplified 10 times with a probe (INR-02, Syntech, Hilversum, the Netherlands), digitally converted through an IDAC-4-USB (Syntech) interface, and visualized and analysed with the software Autospike v. 3.4 (Syntech). A constant flow of 0.65 m/s of humidified air (charcoal-filtered) was delivered through a glass tube to the antenna. The panel of odorants was given to the insect by inserting pipettes containing a piece of filter paper with the correspondent stimulus in a lateral hole of the glass tube and puffing a flow of 2.5 mL of air during 0.5 s through the pipette. For CpomOR1 and CpomOR6a the panel of odorants was prepared by applying 10 μL of a solution of 1.0 μg/μL of the compounds in Supplementary Dataset S2 and Supplementary Dataset S3, for a total amount of 10 μg per stimulus. In the case of CpomOR3, a similar dilution process was used for the dose response experiments of ethyl-(E,Z)-2,4-decadienoate and methyl-(E,Z)-2,4-decadienoate, as well for dose response of experiments of codlemone acetate in CpomOR6a. Compounds were diluted from concentrations ranging from 0.01 ng/μL to 10 μg/μL in decadic steps, allowing reaching concentrations from 100 pg to 100 μg per stimulus when 10 μL of the dilution was applied in the piece of filter paper. In all cases, to characterize the intensity of the response, spike frequency was calculated by subtracting the spikes recorded 0.5 s before the stimulus from the number of spikes recorder 0.5 s after the stimulus and multiplied by 2 to get the response in spikes/s. The number of spikes was corrected accounting for differences in vapour pressure^58^. Dose response experiments between pear ester and its analogue were compared with two-way ANOVA with repeated measures, followed by LSD *post-hoc* test. Dose response experiments of codlemone acetate in CpomOR6a were analysed with one-way ANOVA with repeated measures followed by LSD post-hoc test.

### Source, identity and purity of tested compounds statement

The authors declare that all compounds used in this study were pure according with respective supplier’s standards or sampled from pure stocks used in previously published methods.

## Additional Information

**How to cite this article**: Cattaneo, A. M. *et al*. Candidate pheromone receptors of codling moth *Cydia pomonella* respond to pheromones and kairomones. *Sci. Rep.* **7**, 41105; doi: 10.1038/srep41105 (2017).

**Publisher's note:** Springer Nature remains neutral with regard to jurisdictional claims in published maps and institutional affiliations.

## Supplementary Material

Supplementary Material

## Figures and Tables

**Table 1:** Library of potential codling moth pheromones and synergist compounds screened against CpomOrco + ORs.

Compound	MW (g/mol)	Solubility (M)	LogP	Boiling point (°C at 760 mmHg)	CAS	Source	Reference
(-)-β-caryophyllene	204.35	3.40E-08	6.416 ± 0.248	268.4 ± 10.0	87-44-5	Sigma	1
(E)-β-farnesene	204.35	1.50E-08	6.139 ± 0.304	272.5 ± 20.0	18794-84-8	Bedoukian	1 and 25
(E,E)-8,10-dodecadien-1-yl-acetate (codlemone acetate)	224.34	3.20E-04	5.061 ± 0.223	314.7 ± 11.0	53880-51-6D	Bedoukian Inc	25
(E,E)-8,10-dodecadienol (codlemone)	182.30	2.50E-04	4.096 ± 0.204	270.7 ± 9.0	76600-88-9	Fluka	25
(E,E)-α-farnesene	204.35	1.00E-08	6.304 ± 0.316	279.6 ± 20.0	502-61-4	Bedaukian	1
(Z)-3-hexenol	100.16	0.14	1.697 ± 0.206	156.5 ± 0.0	928-96-1	Aldrich	1
(Z)-3-hexenyl acetate	142.20	0.025	2.400 ± 0.228	174.2 ± 19.0	3681-71-8	Gift from Prof. Peter Witzgall	1
1,8-p-menthadien-7-al (perillaldehyde)	150.22	6.10E-03	3.053 ± 0.335	238.0 ± 29.0	2111-75-3	Gift from Dr. Gigliola Borgonovo	55
1-dodecanol	186.33	5.00E-05	4.914 ± 0.177	258.0 ± 3.0	112-53-8	Sigma Aldrich	25
3-(4-methyl-1-oxopentyl)furan- (perillaketone)	166.22	2.10E-03	2.851 ± 0.318	224.4 ± 13.0	553-84-4	Gift from Dr. Gigliola Borgonovo	55
butyl hexanoate	172.26	2.30E-03	3.842 ± 0.205	206.8 ± 8.0	626-82-4	Bedoukian	25
ethyl-(E,Z)-2,4-decadienoate [(E,Z)-ED]	196.29	1.00E-03	4.454 ± 0.229	264.7 ± 9.0	3025-30-7	Aldrich	1 and 25
Linalool	154.25	6.70E-03	2.795 ± 0.263	198.5 ± 0.0	78-70-6	Firmenich	1
methyl salicilate	152.15	0.021	2.523 ± 0.240	222.0 ± 0.0	119-36-8	Fluka	1
methyl-(E,Z)-2,4-decadienoate [(E,Z)-MD]	182.26	2.30E-03	3.944 ± 0.229	246.0 ± 9.0	4493-42-9	Gift from Prof. Peter Witzgall	33
nonanal	142.24	2.30E-03	3.461 ± 0.223	190.8 ± 3.0	124-19-6	Aldrich	1
(E)-β-ocimene	136.23	2.00E-05	4.418 ± 0.275	175.2 ± 10.0	3779-61-1	Fluka	1

**Table 2:** Primer sequences and melting temperatures.

OR6a 5′-RACE Primer	Sequence	Tm (°C)
5′_OR6a	CCCATGGTACTGCATATACTTCATCACCGAGACG	65.42
**CDS-primers**		
Fw_Orco	ATGATGGGTAAAGTGAAATCTCA	57.60
Rv_Orco	TTACTTCAGTTGTACTAACACCATGA	61.70
Fw_OR6a	ATGCAGACAAAAAGGCAAACCAG	61.00
Rv_OR6a	TTAGTCTGCGAATGTGGCTAGC	61.00
Fw_OR3	ATGTTTAGTTATGAAAATGAAGACAGC	60.80
Rv_OR3	TTAAGTCATTTCTTCAGTAGAGGT	58.30
Fw_OR1	ATGTCTTTGAAAAGCCGTGTTTGG	62.00
Rv_OR1	TTACCCCTCAGCAGCGAAAG	60.50
