# Supplementary material for: Candidate pheromone receptors of codling moth Cydia pomonella respond to pheromones and kairomones
Source: Sci Rep. 2017 Jan 24;7:41105. doi: 10.1038/srep41105 (PMC5259778; doi:10.1038/srep41105)

Candidate pheromone receptors of codling moth *Cydia pomonella* respond to pheromones and kairomones

Alberto Maria Cattaneo, Francisco Gonzalez, Jonas M. Bengtsson, Elizabeth A. Corey, Emmanuelle Jacquin-Joly, Nicolas Montagné, Umberto Salvagnin, William B. Walker III, Peter Witzgall, Gianfranco Anfora and Yuriy V. Bobkov

Supplementary Table S1. Additional list of compounds tested on CpomOrco+ORs in HEK293T.

**Further compounds tested**	**MW (g/mol)**	**Solubility (M)**	**LogP**	**CAS**	**Source**
(-)-carvone	150,22	7.80E-03	2.268±0.334	6485-40-1	MBI lab
(-)-α-pinene	136,23	6,50E-05	4.321±0.237	80-56-8	Fluka
(+)-carvone	150,22	7.80E-03	2.268±0.334	2244-16-8	MBI lab
(+)-nootkatone	218,33	2,40E-04	3.765±0.275	4674-50-4	Givaudan
(E)-2-hexenal	98,14	0,09	1.790±0.281	505-57-7	MBI lab
(E)-2-hexenol	100,16	0,14	1.655±0.212	928-95-0	MBI lab
(*Z*)-3-hexenol	100,16	0,14	1.697±0.206	928-96-1	Safc
(*Z*)-3-hexenyl acetate	142,20	0,025	2.400±0.228	3681-71-8	Safc
(Z)-jasmone	164,24	3,90E-03	2.020±0.337	488-10-8	MBI lab
+/-nerolidol	222,37	2,10E-05	4.682±0.295	3790-78-1	Aldrich
+/-phytol	296,53	8,10E-10	8.230±0.255	150-86-7	Aldrich
1-heptanol	116,20	0,029	2.367±0.177	111-70-6	Aldrich
1-indanone	132,16	4,60E-03	1.419±0.329	83-33-0	Aldrich
1-nonanol	144,25	2,70E-03	3.386±0.177	143-08-8	Fluka
1-octanol	130,23	9,00E-03	2.876±0.177	111-87-5	Sigma Aldrich
1-octen-3-ol	128,21	0,018	2.519±0.220	3391-86-4	MBI lab
1-pentanol	88,15	0,24	1.348±0.176	71-41-0	MBI lab
1-pentene-3-ol	86,13	0,46	0.991±0.220	616-25-1	MBI lab
1-tetradecanol	214,39	2,70E-06	5.933±0.178	112-72-1	Fluka
2-butanone, 4-(4-hydroxyphenyl)- (raspberry ketone)	164,20	0,031	1.309±0.212	5471-51-2	Givaudan
2-propen-1-one, 1-(2-furanyl)-3-(4-methoxyphenyl)-, (2*E*)- (PK analogue 16)	228,24	5,10E-04	2.752±0.338	137444-58-7P	Gift from Dr. Gigliola Borgonovo
2(3H)-furanone, 5-butyldihydro-4-methyl- (whiskey lactone)	156,22	0,011	1.968±0.280	39212-23-2	MBI lab
2,5 dimethyl pyrazine	108,14	6,02	0.687±0.315	123-32-0	MBI lab
2-butyl acetate	116,16	0,081	1.648±0.212	105-46-4	MBI lab
2-ethylfuran	96,13	0,029	2.300±0.241	3208-16-0	MBI lab
2H-pyran, tetrahydro-4-methyl-2-(2-methyl-1-propen-1-yl)-, (2R,4R)- (trans-rose oxide)	154,25	7,40E-03	3.186±0.265	5258-11-7	Givaudan
2H-pyran, tetrahydro-4-methyl-2-(2-methyl-1-propen-1-yl)-, (2S,4R)- (cis-rose oxide)	154,25	7,40E-03	3.186±0.265	3033-23-6	Givaudan
2-heptanone	114,19	0,044	1.996±0.193	110-43-0	MBI lab
2-hexylpyridine	163,26	0,045	3.766±0.188	1129-69-7	MBI lab
2-isobutyl-3-methoxypyrazine	166,22	0,05	2.547±0.377	24683-00-9	MBI lab
2-isobutylthiazole	141,23	0,058	1.715±0.222	18640-74-9	MBI lab
2-methyl-1-butanol	88,15	0,34	1.192±0.187	137-32-6	MBI lab
2-methylbutyl acetate	130,18	0,034	2.158±0.212	624-41-9	MBI lab

2-methylbutyraldehyde	86,13	0,086	1.267±0.227	96-17-3	MBI lab
2-octanone	128,21	0,018	2.506±0.193	111-13-7	MBI lab
2-phenylethanol	122,16	0,16	1.504±0.186	60-12-8	MBI lab
2-propen-1-one, 3-(4-chlorophenyl)-1-(2-furanyl)-, (2*E*)- (PK analogue 18)	232,66	1,20E-04	3.482±0.339	111042-59-2P	Gift from Dr. Gigliola Borgonovo
3-Isothiocyanato-1-propene (Mustard oil)	99,15	3,23E-03	1.9±0.1	55-06-7	Aldrich
3-methyl-1-butanol	88,15	0,34	1.192±0.187	123-51-3	MBI lab
3-methyl-1-pentanol	102,17	0,12	1.702±0.188	589-35-5	MBI lab
3-methyl-2-butenal	84,12	0,31	1.190±0.316	107-86-8	MBI lab
3-octanone	128,21	0,018	2.506±0.193	106-68-3	MBI lab
3-pentanone	86,13	0,26	0.977±0.192	96-22-0	MBI lab
4-ethyl guaiacol (4-ethyl-2-methoxyphenol)	152,19	0,014	2.434±0.224	2785-89-9	Givaudan
4-isopropyl phenol	136,19	0,015	2.986±0.200	99-89-8	Givaudan
4-*tert*-butyl cyclohexanol	156,27	0,01	3.092±0.213	98-52-2	Givaudan
4-tert-butyl cyclohexanone	154,25	5.70E-03	2.630±0.264	98-53-3	Givaudan
4-tert-butyl phenol	150,22	6,30E-03	3.397±0.214	98-54-4	Givaudan
4-vinyl guaiacol (2-methoxy-4-vinylphenol)	150,17	0,015	2.573±0.249	7786-61-0	Givaudan
6-methyl-5-hepten-2-ol	128,21	0,041	2.057±0.236	1569-60-4	MBI lab
6-methyl-5-hepten-2-one	126,20	0.052	1.947±0.238	110-93-0	MBI lab
acetic acid	60,05	3,28	-0.322±0.184	64-19-7	Sigma Aldrich
acetophenone	120,15	0,02	1.674±0.217	98-86-2	Fluka
acetophenone	120,15	0,02	1.674±0.217	98-86-2	MBI lab
acetyl eugenol	206,24	1.40E-03	2.710±0.240	93-28-7	Givaudan
amylbutyrate	158,24	5,40E-03	3.333±0.205	540-18-1	MBI lab
anisole b-cyclocitral	108,14	0,03	2.170±0.203	100-66-3	MBI lab
b-cyclocitral	152,23	6,50E-03	3.100±0.319	432-25-7	Safc
benzaldehyde	106,12	0,02	1.452±0.242	100-52-7	Sigma Aldrich
benzoic acid	122,12	0,046	1.559±0.206	65-85-0	MBI lab
benzothiazole	135,19	0,27	1.899±0.297	95-16-9	MBI lab
benzyl acetate	150,17	0,017	1.998±0.224	140-11-4	MBI lab
benzyl alcohol	108,14	0,043	1.055±0.206	100-51-6	Aldrich
benzyl methyl ether	122,16	0,05	1.843±0.239	538-86-3	Aldrich
b-ionone	192,30	1.20E-3	3.589±0.275	79-77-6	MBI lab
bourgeonal	190,28	4,50E-04	3.486±0.245	18127-01-0	MBI lab
butanol	74,12	0,65	0.839±0.176	71-36-3	MBI lab
butyl acetate	116,16	0,073	1.804±0.205	123-86-4	MBI lab
butyl butanoate	144,21	0,013	2.823±0.205	109-21-7	Aldrich
camphor	152,23	6,90E-03	2.089±0.300	464-49-3	MBI lab
carvacrol	150,22	6,40E-03	3.162±0.205	499-75-2	Aldrich
cineole (1,8-) eucalyptol	154,25	5,90E-03	2.795±0.267	470-82-6	MBI lab
cineole 1,4-	154,25	4,40E-03	2.496±0.266	470-67-7	MBI lab
cinnamaldehyde	132,16	0,023	1.900±0.283	14371-10-9	MBI lab
cis-2-penten-1-ol	86,13	0,4	1.146±0.212	1576-95-0	MBI lab
citral	152,23	0,011	3.127±0.359	5392-40-5	MBI lab
citronellal	154,25	2,90E-03	3.297±0.259	106-23-0	MBI lab
citronellol	156,27	3,00E-03	3.239±0.235	106-22-9	MBI lab

cyclodecanone	154,25	0,013	2.929±0.252	1502-06-3	MBI lab
cycloheptanecarbaldehyde	126,20	0,012	2.394±0.225	4277-29-6	MBI lab
cyclohexanone	98,14	0.15	0.821±0.251	108-94-1	MBI lab
cyclopentanecarboxaldehyde	98,14	0,041	1.339±0.225	872-53-7	MBI lab
d-decalactone	170,25	5.60E-03	2.469±0.278	705-86-2	MBI lab
decanal	156,27	9,80E-04	3.970±0.223	112-31-2	Sigma
dihydro eugenol (2-methoxy-4-propylphenol)	166,22	6,00E-03	2.943±0.224	2785-87-7	Givaudan
4-Allylanisole (estragol)	148,20	4,30E-03	3.088±0.223	140-67-0	Aldrich
ethyl vanillin	166,17	9,80E-03	1.718±0.272	121-32-4	Givaudan
eugenol	164,20	0,011	2.403±0.236	97-53-0	Aldrich
farnesol	222,37	1,90E-05	4.828±0.309	4602-84-0	Aldrich
fennaldehyde	178,23	3,50E-03	2.023±0.250	5462-06-6	Givaudan
geraniol	154,25	5,90E-03	2.942±0.271	106-24-1	Aldrich
geranylacetone	194,31	2,30E-03	3.834±0.268	3796-70-1	MBI lab
hedione	226,31	1,80E-03	2.653±0.272	24851-98-7	MBI lab
helional	192,21	8,10E-04	1.982±0.343	1205-17-0	MBI lab
heliotropin	150,13	4,40E-03	1.050±0.302	120-57-0	Givaudan
heptanal	114,19	0,013	2.442±0.223	111-71-7	MBI lab
heptyl butyrate	186,29	1,00E-03	4.352±0.206	5870-93-9	MBI lab
hexanal	100,16	0,031	1.932±0.223	66-25-1	MBI lab
hexanol	102,17	0,086	1.858±0.177	111-27-3	Acros
hexyl 2-methylbutanoate	186,29	1,10E-03	4.196±0.212	10032-15-2	Safc
hexyl acetate	144,21	0,013	2.823±0.205	142-92-7	MBI lab
hexyl alcohol	102,17	0,086	1.858±0.177	111-27-3	MBI lab
hexyl hexanoate	200,32	4,40E-04	4.861±0.206	6378-65-0	Safc
isoamylacetate	130,18	0,034	2.158±0.212	123-92-2	MBI lab
isobutyl acetate	116,16	0,081	1.648±0.212	110-19-0	MBI lab
isoeugenol	164,20	7,30E-03	3.081±0.248	97-54-1	Givaudan
isomenthone	154,25	5,50E-03	2.755±0.260	491-07-6	MBI lab
isopentyl acetate	130,18	0,034	2.158±0.212	123-92-2	Fluka
isosafrol	162,19	5,90E-04	3.904±0.349	120-58-1	Givaudan
isovaleraldehyde	86,13	0.086	1.267±0.227	590-86-3	MBI lab
isovaleric acid	102,13	0,23	1.051±0.193	503-74-2	MBI lab
isovaleronitrile	83,13	0,11	1.039±0.199	625-28-5	MBI lab
lilial	204,31	2,20E-04	3.839±0.249	80-54-6	MBI lab
limonene, R(+)	136,23	2,50E-05	4.552±0.241	5989-27-5	Aldrich
linalool	154,25	6,70E-03	2.795±0.263	78-70-6	Aldrich
liral	210,31	3,10E-03	2.424±0.256	31906-04-4	MBI lab
menthone (-)	154,25	5,50E-03	2.755±0.260	89-80-5	MBI lab
methional	104,17	0.26	0.436±0.323	3268-49-3	MBI lab
methyl benzoate	136,15	0,02	2.124±0.204	93-58-3	MBI lab
methyl diantilis	182,22	0,039	1.571±0.265	5595-79-9	Givaudan
methyl eugenol	178,23	4,30E-03	2.655±0.243	93-15-2	Givaudan
methyl valerate	116,16	0,073	1.804±0.205	624-24-8	MBI lab
methyl-iso-eugenol	178,23	3.60E-03	3.049±0.239	93-16-3	Givaudan
methyl atratate, evernyl (mousse cristal)	196,20	0,01	2.843±0.336	4707-47-5	Givaudan
n-butyl acetate	116,16	0,073	1.804±0.205	123-86-4	MBI lab
nonanedeioic acid (azelaic acid)	188,22	0,046	1.196±0.197	123-99-9	MBI lab

octanal	128,21	5.40E-03	2.951±0.223	124-13-0	MBI lab
octanoic acid	144,21	0,015	2.735±0.184	124-07-2	MBI lab
orivone 4-(tert.-pentyl)-cyclohexanone	168,28	2.40E-03	3.140±0.264	16587-71-6	Givaudan
p-cymene	134,22	9,90E-05	4.014±0.189	99-87-6	MBI lab
pentanal = valeraldehyde	86,13	0,077	1.423±0.222	110-62-3	MBI lab
pentanol	88,15	0,24	1.348±0.176	71-41-0	MBI lab
pentyl acetate (amyl acetate)	130,18	0.030	2.314±0.205	628-63-7	MBI lab
phenyl acetaldehyde	120,15	0,016	1.760±0.224	122-78-1	Aldrich
phenylacetaldehyde	120,15	0,016	1.760±0.224	122-78-1	MBI lab
phenylethylamine	121,18	0,085	1.435±0.189	64-04-0	MBI lab
prenyl acetate	128,17	0,069	2.017±0.274	1191-16-8	MBI lab
propan-2-ol (isopropanol)	60,10	2,34	0.173±0.187	67-63-0	MBI lab
propyl acetate	102,13	0.18	1.295±0.205	109-60-4	MBI lab
propyl hexanoate	158,24	5,40E-03	3.333±0.205	626-77-7	Safc
pyrazine	80,09	12,5	-0.002±0.232	290-37-9	MBI lab
pyrollidine	71,12	2,15	0.085±0.242	123-75-1	MBI lab
thymol	150,22	5,80E-03	3.252±0.205	89-83-8	Sigma
trans-2-heptenal	112,17	0,037	2.300±0.282	18829-55-5	MBI lab
trans-2-hexenal	98,14	0,09	1.790±0.281	6728-26-3	Aldrich
trans-2-pentenal	84,12	0,22	1.281±0.281	1576-87-0	MBI lab
trans-2-hexen-1-ol	100,16	0,14	1.697±0.206	928-97-2	Sigma Aldrich
triethylamine	101,19	0,31	1.647±0.222	121-44-8	MBI lab
tropional	192,21	8,10E-04	1.982±0.343	1205-17-0	Givaudan
undecanal	170,29	4.20E-04	4.480±0.223	112-44-7	MBI lab
vanillin	152,15	0,024	1.208±0.272	121-33-5	Givaudan
vanillyl acetone (zingerone)	194,23	0,024	1.168±0.237	122-48-5	Givaudan
α-humulene	204,35	1,10E-08	6.592±0.249	6753-98-6	Fluka
γ-decalactone	170,25	4.20E-03	2.451±0.278	706-14-9	MBI lab

Candidate pheromone receptors of codling moth *Cydia pomonella* respond to pheromones and kairomones

Alberto Maria Cattaneo, Francisco Gonzalez, Jonas M. Bengtsson, Elizabeth A. Corey, Emmanuelle Jacquin-Joly, Nicolas Montagné, Umberto Salvagnin, William B. Walker III, Peter Witzgall, Gianfranco Anfora and Yuriy V. Bobkov

Supplementary Dataset S2. Responses of CpomOR6a expressed in *Drosophila* at1 OSNs to pear ester, codlemone, codlemone acetate and structurally related compounds.

Replicate	Compound	Spike/s
1	Hexane	-2
	Dodecadien-1-yl acetate (12Ac)	4
	(E)-8-dodecadien-1-yl acetate [(E)-8:12Ac]	10
	(Z)-8-dodecadien-1yl acetate [(Z)-8:12Ac]	0
	(E)-10-dodecadien-1-yl acetate [(E)-10:12Ac]	26
	(Z)-10-dodecadien-1-yl acetate [(Z)-10:12Ac]	0
	(E,E)-8,10-dodecadien-1-ol [(E,E)-8,10:12OH]	0
	(Z,Z)-8,10-dodecadien-1yl acetate [(Z,Z)-8,10:12Ac]	14
	(Z,E)-8,10-dodecadien-1yl acetate [(Z,E)-8,10:12Ac]	10
	(E,Z)-8,10-dodecadien-1yl acetate [(E,Z)-8,10:12Ac]	6
	(E,E)-8,10-dodecadien-1yl acetate [(E,E)-8,10:12Ac]	46
	Ethyl-(E,Z)-2,4-decadienoate [(E,Z)-ED]	2
2	Hexane	2
	Dodecadien-1-yl acetate (12Ac)	4
	(E)-8-dodecadien-1-yl acetate [(E)-8:12Ac]	0
	(Z)-8-dodecadien-1yl acetate [(Z)-8:12Ac]	0
	(E)-10-dodecadien-1-yl acetate [(E)-10:12Ac]	26
	(Z)-10-dodecadien-1-yl acetate [(Z)-10:12Ac]	6
	(E,E)-8,10-dodecadien-1-ol [(E,E)-8,10:12OH]	0
	(Z,Z)-8,10-dodecadien-1yl acetate [(Z,Z)-8,10:12Ac]	12
	(Z,E)-8,10-dodecadien-1yl acetate [(Z,E)-8,10:12Ac]	2
	(E,Z)-8,10-dodecadien-1yl acetate [(E,Z)-8,10:12Ac]	10
	(E,E)-8,10-dodecadien-1yl acetate [(E,E)-8,10:12Ac]	50
	Ethyl-(E,Z)-2,4-decadienoate [(E,Z)-ED]	-2
3	Hexane	2
	Dodecadien-1-yl acetate (12Ac)	-2
	(E)-8-dodecadien-1-yl acetate [(E)-8:12Ac]	4
	(Z)-8-dodecadien-1yl acetate [(Z)-8:12Ac]	0
	(E)-10-dodecadien-1-yl acetate [(E)-10:12Ac]	28
	(Z)-10-dodecadien-1-yl acetate [(Z)-10:12Ac]	2
	(E,E)-8,10-dodecadien-1-ol [(E,E)-8,10:12OH]	-4
	(Z,Z)-8,10-dodecadien-1yl acetate [(Z,Z)-8,10:12Ac]	10

	(Z,E)-8,10-dodecadien-1yl acetate [(Z,E)-8,10:12Ac]	-4
	(E,Z)-8,10-dodecadien-1yl acetate [(E,Z)-8,10:12Ac]	16
	(E,E)-8,10-dodecadien-1yl acetate [(E,E)-8,10:12Ac]	44
	Ethyl-(E,Z)-2,4-decadienoate [(E,Z)-ED]	8
4	Hexane	-2
	Dodecadien-1-yl acetate (12Ac)	6
	(E)-8-dodecadien-1-yl acetate [(E)-8:12Ac]	4
	(Z)-8-dodecadien-1yl acetate [(Z)-8:12Ac]	4
	(E)-10-dodecadien-1-yl acetate [(E)-10:12Ac]	60
	(Z)-10-dodecadien-1-yl acetate [(Z)-10:12Ac]	2
	(E,E)-8,10-dodecadien-1-ol [(E,E)-8,10:12OH]	6
	(Z,Z)-8,10-dodecadien-1yl acetate [(Z,Z)-8,10:12Ac]	16
	(Z,E)-8,10-dodecadien-1yl acetate [(Z,E)-8,10:12Ac]	8
	(E,Z)-8,10-dodecadien-1yl acetate [(E,Z)-8,10:12Ac]	6
	(E,E)-8,10-dodecadien-1yl acetate [(E,E)-8,10:12Ac]	54
	Ethyl-(E,Z)-2,4-decadienoate [(E,Z)-ED]	2
5	Hexane	4
	Dodecadien-1-yl acetate (12Ac)	10
	(E)-8-dodecadien-1-yl acetate [(E)-8:12Ac]	12
	(Z)-8-dodecadien-1yl acetate [(Z)-8:12Ac]	-2
	(E)-10-dodecadien-1-yl acetate [(E)-10:12Ac]	34
	(Z)-10-dodecadien-1-yl acetate [(Z)-10:12Ac]	6
	(E,E)-8,10-dodecadien-1-ol [(E,E)-8,10:12OH]	0
	(Z,Z)-8,10-dodecadien-1yl acetate [(Z,Z)-8,10:12Ac]	20
	(Z,E)-8,10-dodecadien-1yl acetate [(Z,E)-8,10:12Ac]	0
	(E,Z)-8,10-dodecadien-1yl acetate [(E,Z)-8,10:12Ac]	8
	(E,E)-8,10-dodecadien-1yl acetate [(E,E)-8,10:12Ac]	80
	Ethyl-(E,Z)-2,4-decadienoate [(E,Z)-ED]	-14
6	Hexane	16
	Dodecadien-1-yl acetate (12Ac)	6
	(E)-8-dodecadien-1-yl acetate [(E)-8:12Ac]	26
	(Z)-8-dodecadien-1yl acetate [(Z)-8:12Ac]	22
	(E)-10-dodecadien-1-yl acetate [(E)-10:12Ac]	36
	(Z)-10-dodecadien-1-yl acetate [(Z)-10:12Ac]	12
	(E,E)-8,10-dodecadien-1-ol [(E,E)-8,10:12OH]	2
	(Z,Z)-8,10-dodecadien-1yl acetate [(Z,Z)-8,10:12Ac]	20
	(Z,E)-8,10-dodecadien-1yl acetate [(Z,E)-8,10:12Ac]	18
	(E,Z)-8,10-dodecadien-1yl acetate [(E,Z)-8,10:12Ac]	24
	(E,E)-8,10-dodecadien-1yl acetate [(E,E)-8,10:12Ac]	46
	Ethyl-(E,Z)-2,4-decadienoate [(E,Z)-ED]	8
	Hexane	8

7	Dodecadien-1-yl acetate (12Ac)	0
	(E)-8-dodecadien-1-yl acetate [(E)-8:12Ac]	30
	(Z)-8-dodecadien-1yl acetate [(Z)-8:12Ac]	32
	(E)-10-dodecadien-1-yl acetate [(E)-10:12Ac]	50
	(Z)-10-dodecadien-1-yl acetate [(Z)-10:12Ac]	14
	(E,E)-8,10-dodecadien-1-ol [(E,E)-8,10:12OH]	14
	(Z,Z)-8,10-dodecadien-1yl acetate [(Z,Z)-8,10:12Ac]	10
	(Z,E)-8,10-dodecadien-1yl acetate [(Z,E)-8,10:12Ac]	26
	(E,Z)-8,10-dodecadien-1yl acetate [(E,Z)-8,10:12Ac]	16
	(E,E)-8,10-dodecadien-1yl acetate [(E,E)-8,10:12Ac]	82
	Ethyl-(E,Z)-2,4-decadienoate [(E,Z)-ED]	4
8	Hexane	16
	Dodecadien-1-yl acetate (12Ac)	12
	(E)-8-dodecadien-1-yl acetate [(E)-8:12Ac]	30
	(Z)-8-dodecadien-1yl acetate [(Z)-8:12Ac]	6
	(E)-10-dodecadien-1-yl acetate [(E)-10:12Ac]	66
	(Z)-10-dodecadien-1-yl acetate [(Z)-10:12Ac]	4
	(E,E)-8,10-dodecadien-1-ol [(E,E)-8,10:12OH]	0
	(Z,Z)-8,10-dodecadien-1yl acetate [(Z,Z)-8,10:12Ac]	32
	(Z,E)-8,10-dodecadien-1yl acetate [(Z,E)-8,10:12Ac]	40
	(E,Z)-8,10-dodecadien-1yl acetate [(E,Z)-8,10:12Ac]	34
	(E,E)-8,10-dodecadien-1yl acetate [(E,E)-8,10:12Ac]	62
	Ethyl-(E,Z)-2,4-decadienoate [(E,Z)-ED]	-2
9	Hexane	4
	Dodecadien-1-yl acetate (12Ac)	-12
	(E)-8-dodecadien-1-yl acetate [(E)-8:12Ac]	10
	(Z)-8-dodecadien-1yl acetate [(Z)-8:12Ac]	-8
	(E)-10-dodecadien-1-yl acetate [(E)-10:12Ac]	26
	(Z)-10-dodecadien-1-yl acetate [(Z)-10:12Ac]	4
	(E,E)-8,10-dodecadien-1-ol [(E,E)-8,10:12OH]	2
	(Z,Z)-8,10-dodecadien-1yl acetate [(Z,Z)-8,10:12Ac]	12
	(Z,E)-8,10-dodecadien-1yl acetate [(Z,E)-8,10:12Ac]	10
	(E,Z)-8,10-dodecadien-1yl acetate [(E,Z)-8,10:12Ac]	2
	(E,E)-8,10-dodecadien-1yl acetate [(E,E)-8,10:12Ac]	58
	Ethyl-(E,Z)-2,4-decadienoate [(E,Z)-ED]	14

Candidate pheromone receptors of codling moth *Cydia pomonella* respond to pheromones and kairomones

Alberto Maria Cattaneo, Francisco Gonzalez, Jonas M. Bengtsson, Elizabeth A. Corey, Emmanuelle Jacquin-Joly, Nicolas Montagné, Umberto Salvagnin, William B. Walker III, Peter Witzgall, Gianfranco Anfora and Yuriy V. Bobkov

Supplementary Dataset S3. Responses of CpomOR1 expressed in *Drosophila* at1 OSNs to pheromones, synergists and their combinations.

Replicate	Compound(s)	Spikes/s
1	Blank	4
	Hexane	-4
	(E8,E10)-12:OH	6
	(E8,Z10)-12:OH	-2
	(Z8,E10)-12:OH	0
	(Z8,Z10)-12:OH	-2
	(E8,E10)-12:Ac	2
	(E8)-12:OH	-2
	(E9)-12:OH	-4
	(E10)-12:OH	-2
	12:OH	-2
	(E)-β-Farnesene	0
	Butyl hexanoate	4
	Ethyl-(E2,Z4)-decadienoate	-2
	(Z9,E12)-14:Ac	2
	4,8-Dimethyl-1,(E)-3,7-nonatriene	2
	3,7-Dimethyl-1,(E)-3,6-octatriene	2
	(E8,E10)-12:OH +(E)-β-Farnesene	-2
	(E8,E10)-12:OH + Butyl hexanoate	2
	(E8,E10)-12:OH +Ethyl-(E2,Z4)-decadienoate	6
	(E8,E10)-12:OH +(Z9,E12)- 14:Ac	2
	(E8,E10)-12:OH + 4,8-Dimethyl-1,(E)-3,7-nonatriene	4
	(E8,E10)-12:OH + 3,7-Dimethyl-1,(E)-3,6-octatriene	2
	(E8,E10)-12:OH (100 ng)	-4
2	Blank	-6
	Hexane	0
	(E8,E10)-12:OH	2
	(E8,Z10)-12:OH	-2
	(Z8,E10)-12:OH	0
	(Z8,Z10)-12:OH	0
	(E8,E10)-12:Ac	4
	(E8)-12:OH	4
	(E9)-12:OH	0
	(E10)-12:OH	-2
	12:OH	4

	(E)-β-Farnesene	4
	Butyl hexanoate	-4
	Ethyl-(E2,Z4)-decadienoate	2
	(Z9,E12)-14:Ac	-4
	4,8-Dimethyl-1,(E)-3,7-nonatriene	-6
	3,7-Dimethyl-1,(E)-3,6-octatriene	8
	(E8,E10)-12:OH +(E)-β-Farnesene	2
	(E8,E10)-12:OH + Butyl hexanoate	-2
	(E8,E10)-12:OH +Ethyl-(E2,Z4)-decadienoate	-2
	(E8,E10)-12:OH +(Z9,E12)- 14:Ac	-4
	(E8,E10)-12:OH + 4,8-Dimethyl-1,(E)-3,7-nonatriene	-2
	(E8,E10)-12:OH + 3,7-Dimethyl-1,(E)-3,6-octatriene	2
	(E8,E10)-12:OH (100 ng)	2
3	Blank	-2
	Hexane	0
	(E8,E10)-12:OH	0
	(E8,Z10)-12:OH	0
	(Z8,E10)-12:OH	6
	(Z8,Z10)-12:OH	0
	(E8,E10)-12:Ac	4
	(E8)-12:OH	0
	(E9)-12:OH	2
	(E10)-12:OH	0
	12:OH	2
	(E)-β-Farnesene	2
	Butyl hexanoate	2
	Ethyl-(E2,Z4)-decadienoate	2
	(Z9,E12)-14:Ac	2
	4,8-Dimethyl-1,(E)-3,7-nonatriene	2
	3,7-Dimethyl-1,(E)-3,6-octatriene	0
	(E8,E10)-12:OH +(E)-β-Farnesene	0
	(E8,E10)-12:OH + Butyl hexanoate	-6
	(E8,E10)-12:OH +Ethyl-(E2,Z4)-decadienoate	-2
	(E8,E10)-12:OH +(Z9,E12)- 14:Ac	-4
	(E8,E10)-12:OH + 4,8-Dimethyl-1,(E)-3,7-nonatriene	0
	(E8,E10)-12:OH + 3,7-Dimethyl-1,(E)-3,6-octatriene	-2
	(E8,E10)-12:OH (100 ng)	2
4	Blank	-4
	Hexane	-8
	(E8,E10)-12:OH	-12
	(E8,Z10)-12:OH	-2
	(Z8,E10)-12:OH	8
	(Z8,Z10)-12:OH	4
	(E8,E10)-12:Ac	2

	(E8)-12:OH	0
	(E9)-12:OH	4
	(E10)-12:OH	-4
	12:OH	-2
	(E)-β-Farnesene	0
	Butyl hexanoate	0
	Ethyl-(E2,Z4)-decadienoate	2
	(Z9,E12)-14:Ac	-10
	4,8-Dimethyl-1,(E)-3,7-nonatriene	4
	3,7-Dimethyl-1,(E)-3,6-octatriene	2
	(E8,E10)-12:OH +(E)-β-Farnesene	-6
	(E8,E10)-12:OH + Butyl hexanoate	2
	(E8,E10)-12:OH +Ethyl-(E2,Z4)-decadienoate	-8
	(E8,E10)-12:OH +(Z9,E12)- 14:Ac	8
	(E8,E10)-12:OH + 4,8-Dimethyl-1,(E)-3,7-nonatriene	0
	(E8,E10)-12:OH + 3,7-Dimethyl-1,(E)-3,6-octatriene	-2
	(E8,E10)-12:OH (100 ng)	-8
5	Blank	6
	Hexane	-4
	(E8,E10)-12:OH	0
	(E8,Z10)-12:OH	-2
	(Z8,E10)-12:OH	-4
	(Z8,Z10)-12:OH	12
	(E8,E10)-12:Ac	4
	(E8)-12:OH	2
	(E9)-12:OH	-2
	(E10)-12:OH	2
	12:OH	2
	(E)-β-Farnesene	2
	Butyl hexanoate	-8
	Ethyl-(E2,Z4)-decadienoate	10
	(Z9,E12)-14:Ac	0
	4,8-Dimethyl-1,(E)-3,7-nonatriene	-8
	3,7-Dimethyl-1,(E)-3,6-octatriene	4
	(E8,E10)-12:OH +(E)-β-Farnesene	4
	(E8,E10)-12:OH + Butyl hexanoate	6
	(E8,E10)-12:OH +Ethyl-(E2,Z4)-decadienoate	-12
	(E8,E10)-12:OH +(Z9,E12)- 14:Ac	0
	(E8,E10)-12:OH + 4,8-Dimethyl-1,(E)-3,7-nonatriene	4
	(E8,E10)-12:OH + 3,7-Dimethyl-1,(E)-3,6-octatriene	10
	(E8,E10)-12:OH (100 ng)	-4

Candidate pheromone receptors of codling moth Cydia pomonella respond to pheromones and kairomones

Alberto Maria Cattaneo, Francisco Gonzalez, Jonas M. Bengtsson, Elizabeth A. Corey, Emmanuelle Jacquin-Joly, Nicolas Montagné, Umberto Salvagnin, William B. Walker III, Peter Witzgall, Gianfranco Anfora and Yuriy V. Bobkov

Supplementary Figure S4. Response of CpomOrco+OR6a transfected HEK293T to (E,E)-codlemone acetate [(E,E)-8,10:12Ac] and codlemone [(E,E)-8,10:12OH]. Left: comparison between amplitudes of the calcium responses (mean of the maximum response ± SEM) to VUAA3 100 µM (37.96 ± 20.98, dF; above), codlemone 50 µM (0.38 ± 1.84, dF; middle) and codlemone acetate 50 µM (12.04 ± 8.17, dF; below); n = 70. Right: comparison between amplitudes of the calcium responses (mean of the maximum response ± SEM) to VUAA3 250 µM (69.71 ± 27.29, dF; above), codlemone 1500 µM (5.7 ± 3.64, dF; middle) and codlemone acetate 1500 µM (18.91 ± 10.31, dF; below); n = 68.

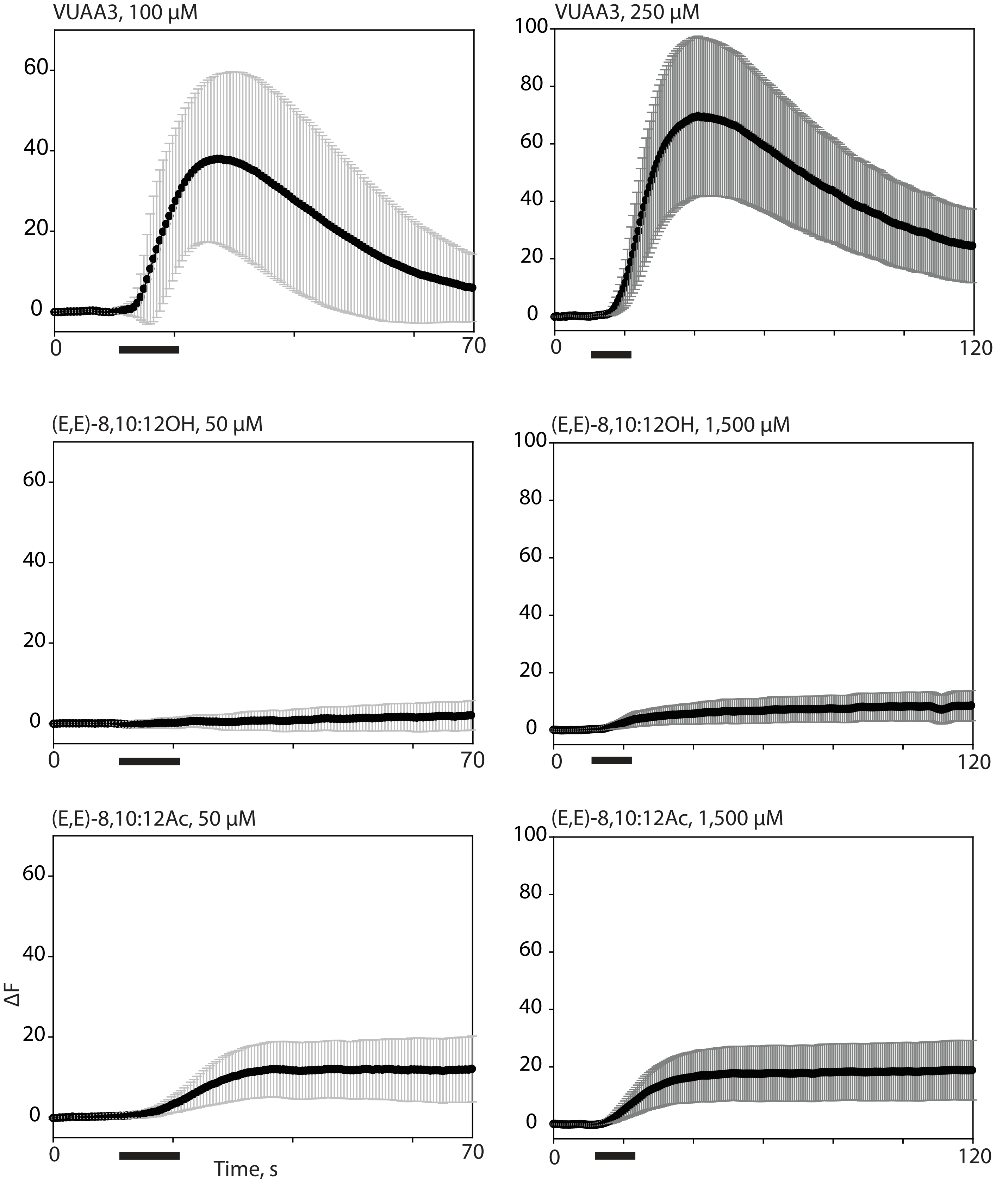

Candidate pheromone receptors of codling moth Cydia pomonella respond to pheromones and kairomones

Alberto Maria Cattaneo, Francisco Gonzalez, Jonas M. Bengtsson, Elizabeth A. Corey, Emmanuelle Jacquin-Joly, Nicolas Montagné, Umberto Salvagnin, William B. Walker III, Peter Witzgall, Gianfranco Anfora and Yuriy V. Bobkov

Supplementary Figure S5. Spike trains from Single Sensillum Recordings of Drosophila at1 OSNs expressing CpomOR6a when stimulated with Hexane and 10 µg doses of (E,Z)-8,10:12Ac, (Z,Z)-8,10:12Ac, (E)-10:12Ac and (E,E)-8,10:12Ac. Black bar: stimulus (0.5 s).

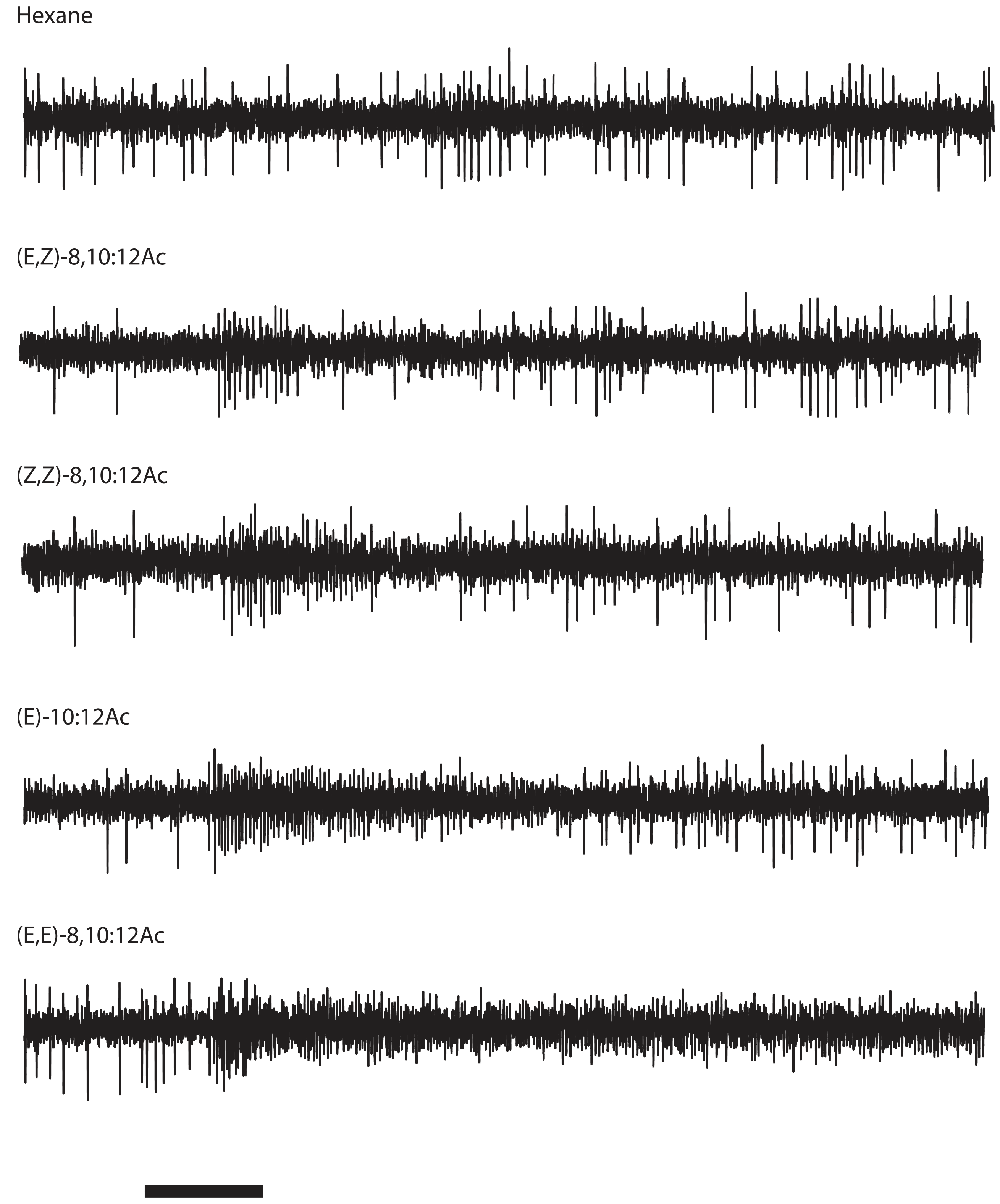

Supplement: Supplementary Material [file srep41105-s1.pdf]
